# Host-parasite co-metabolic activation of antitrypanosomal aminomethyl-benzoxaboroles

**DOI:** 10.1371/journal.ppat.1006850

**Published:** 2018-02-09

**Authors:** Ning Zhang, Martin Zoltner, Ka-Fai Leung, Paul Scullion, Sebastian Hutchinson, Ricardo C. del Pino, Isabel M. Vincent, Yong-Kang Zhang, Yvonne R. Freund, Michael R. K. Alley, Robert T. Jacobs, Kevin D. Read, Michael P. Barrett, David Horn, Mark C. Field

**Affiliations:** 1 Wellcome Centre for Anti-Infectives Research, School of Life Sciences, University of Dundee, Dundee, United Kingdom; 2 Department of Pathology, University of Cambridge, Cambridge, United Kingdom; 3 Wellcome Centre for Molecular Parasitology, Institute of Infection, Immunity and Inflammation, University of Glasgow, Glasgow, United Kingdom; 4 Anacor Pharmaceuticals, Inc., Palo Alto, California, United States of America; U Tex SouthWestern, UNITED STATES

## Abstract

Recent development of benzoxaborole-based chemistry gave rise to a collection of compounds with great potential in targeting diverse infectious diseases, including human African Trypanosomiasis (HAT), a devastating neglected tropical disease. However, further medicinal development is largely restricted by a lack of insight into mechanism of action (MoA) in pathogenic kinetoplastids. We adopted a multidisciplinary approach, combining a high-throughput forward genetic screen with functional group focused chemical biological, structural biology and biochemical analyses, to tackle the complex MoAs of benzoxaboroles in *Trypanosoma brucei*. We describe an oxidative enzymatic pathway composed of host semicarbazide-sensitive amine oxidase and a trypanosomal aldehyde dehydrogenase TbALDH3. Two sequential reactions through this pathway serve as the key underlying mechanism for activating a series of 4-aminomethylphenoxy-benzoxaboroles as potent trypanocides; the methylamine parental compounds as pro-drugs are transformed first into intermediate aldehyde metabolites, and further into the carboxylate metabolites as effective forms. Moreover, comparative biochemical and crystallographic analyses elucidated the catalytic specificity of TbALDH3 towards the benzaldehyde benzoxaborole metabolites as xenogeneic substrates. Overall, this work proposes a novel drug activation mechanism dependent on both host and parasite metabolism of primary amine containing molecules, which contributes a new perspective to our understanding of the benzoxaborole MoA, and could be further exploited to improve the therapeutic index of antimicrobial compounds.

## Author summary

Human African Trypanomiasis (HAT) is among a list of Neglected Tropical Diseases (NTDs) that impose devastating burdens on both public health and economy of some of the most unprivileged societies across the world. To secure the long-term global control of the disease, it is critical to understand the mechanisms underlying the interactions of drugs and drug candidates with the causative agents as well as resistance potentially arising from use of the compounds. We demonstrated here a metabolic enzymatic cascade dependent on a host-pathogen interaction that determines potency against *T*. *brucei* of a series of benzoxaborole compounds. More importantly, this pathway represents a metabolic interaction network between host and pathogen, illuminating an important perspective on understanding mechanism of action.

## Introduction

Encouraged by significant advances in the disease control since the close of the 20^th^ century, the World Health Organization (WHO) targeted HAT for eradication by 2020 [1]. A pressing obstacle to achieving this goal is the limited and obsolete range of treatments available that are compromised by deleterious side-effects, poor oral bioavailability and an alarming increase in drug resistance in the field [2–5].

Several new candidate drugs have advanced through the development pipeline, including acoziborole (SCYX-7158/AN5568) [6], a lead compound currently in a phase 2/3 clinical trial, and AN7973/SCYX-1608210 and AN7119/SCYX1330682 as back-ups [7, 8]. These compounds represent a class of hemiboronic acids with distinctive chemical and pharmacological features [9, 10]. The cyclic boronic ester in the molecules provides a good balance between the Lewis acidity essential for forming interactions with biochemical targets and the physicochemical properties important for good bioavailability. Molecular insights into the mechanism of action (MoA) in pathogenic model organisms has greatly contributed to development and strategies for assessing potential risk of resistance for this series of molecules. However, the benzoxaborole core structure is highly adaptive to substitution of function groups, which not only contributes to great chemical diversity but also gives rise to a broad MoA spectrum. The latter is manifested in a variety of targets and efficacy factors proposed from study of benzoxaboroles in various diseases and conditions, including proteases, phophodiesterases, kinases, anhydrolases, aminoacyl-tRNA synthetases, reductases and RNA splicing factors [11–20]. Furthermore, how the uptake and metabolism of these compounds occur in the context of infections remains as a significant gap in our understanding of benxoxaboroles. An earlier study examining the impact of resistance to acoziborole, together with identification of possible interacting proteins was inconclusive with regard to MoA [21].

Here, we adopted an approach combining forward genetics, biochemistry and structural biology and identified a metabolic pathway critical for achieving the trypanocidal activity of a series of 4-aminomethylphenoxy benzoxaboroles. The pathway involves two oxidation reactions occurring sequentially in the host and the parasite. This highlights the importance of metabolic interaction between host and pathogen [22–25] in considering novel MoAs, and contributes to our improved understanding of benzoxaborole MoA.

## Results

### Genetic components that sensitize trypanosomes to 4-aminomethylphenoxy-benzoxaborole

**Fig 1:**
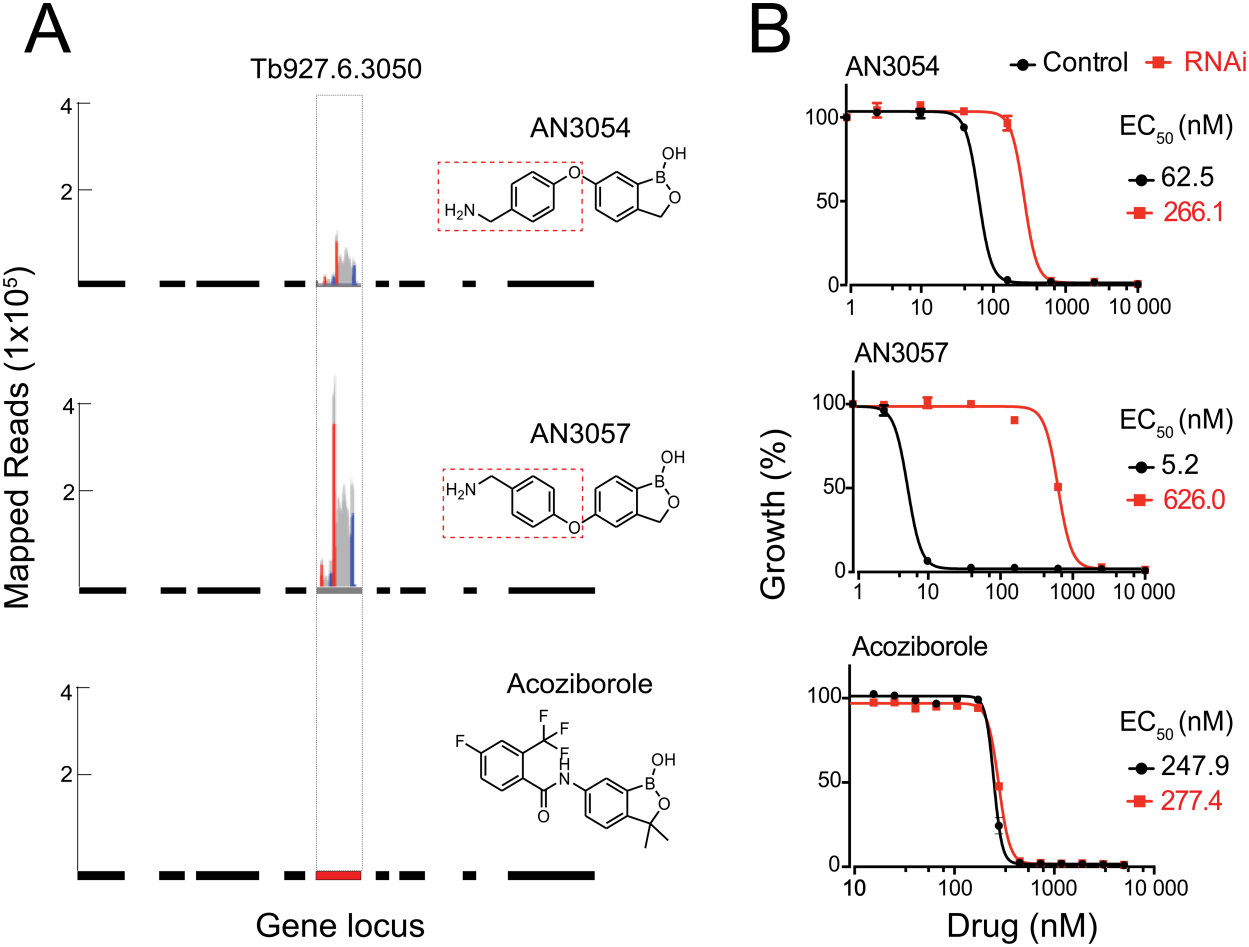
Tb927.6.3050 serves as a specific potency determinant for aminomethylphenoxy benzoxaboroles. (**A**) Tb927.6.3050 identification in RIT-seq from a genome-scale screening for potency determinants of benzoxaboroles. The gene locus is indicated in red, with flanking genes in black, in the relevant chromosomal context. Each peak represents an identification by sequencing and the relative height corresponds to the number of reads. A pair of short sequences were introduced in the original library [26], flanking the individual RNA*i* targeting fragment in both directions, as a unique bar code to ensure sequencing specificity. The corresponding identifications are indicated in color, red for the forward sequence and blue for the reverse, distinguished from all other identification indicated in grey. The aminomethylphenoxy moieties in the respective compounds are indicated with the dotted rectangles. (**B**) The changes in potency (EC_50_) of chemically diverse benzoxaboroles upon Tb927.6.3050 RNA*i*.

We screened a set of benzoxoaboroles with variable substituents against a genome-scale RNA*i* library in *T*. *brucei* [26, 27] to uncover the genetic factors that sensitize trypanosomes to the compounds. These compounds included AN3054 and AN3057 that share a 4-aminomethylphenoxy substituent linked via the 6- or 5-position of the benzoxaborole core, and acoziborole that contains a 6-carboxyamide substituent (Fig 1A). We identified a high confidence hit, Tb927.6.3050, specifically with the 4-aminomethylphenoxy derivatives (AN3054 and AN3057), in addition to a cohort of candidates determining the sensitivity of the parasites towards a broad range of benzoxaboroles (unpublished data).

We then examined the impact of Tb927.6.3050 RNA*i* on the sensitivity of trypanosomes to individual compounds (Fig 1B). Upon knockdown, the trypanosomes were significantly desensitized to either AN3054 or AN3057 as opposed to acoziborole. Moreover, there was a further divergence in the impact of the knockdown between AN3054 and AN3057, with more profound impact on the latter. This is also consistent with the results from the initial genetics screens where the signal for Tb927.6.3050 was more pronounced in the screen with AN3057 than with AN3054 (Fig 1A). Taken together, these data suggest a specific structure-activity relationship (SAR) between Tb927.6.3050 and 4-aminomethylphenoxy derivatives.

### The SAR between Tb927.6.5030 and 4-aminomethylphenoxy substituents

**Fig 2:**
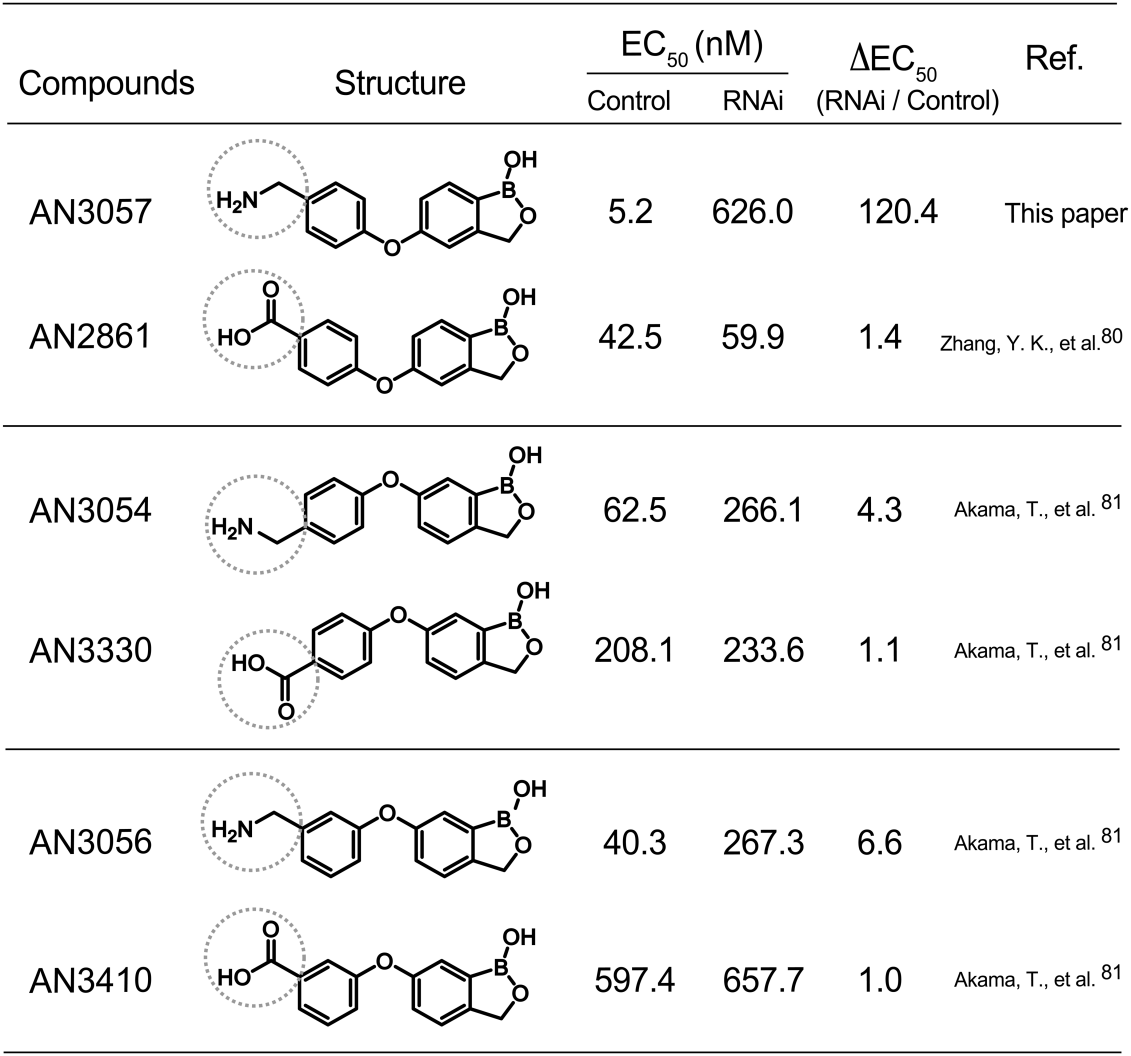
Potencies of the phenoxy derivatives upon Tb927.6.3050 RNAi. The compounds are listed in pair according to structural similarity in the functional groups. The methylamine and carboxylic acid groups are highlighted with dot circles. Ref. stands for reference.

To define this SAR, we first investigated the correlation between the potency of related but distinctive phenoxy compounds and Tb927.6.3050 RNA*i*. Included were three aminomethylphenoxy-substitution (-CH_2_NH_2_) isomers, i.e. AN3057, AN3054 and AN3056, and three carboxyphenoxy-substitution (-CO_2_H) isomers, i.e. AN2861, AN3330 and AN3410, with each aminomethylphenoxy isomer paired with the corresponding carboxyphenoxy isomer as shown in Fig 2. The potencies of all three aminomethylphenoxy isomers were significantly compromised by the knockdown, in contrast to the impact of the carboxyphenoxy equivalents. Thus, the methylamine moiety shared by the aminomethylphenoxy derivatives is the key link to the function of Tb927.6.3050 in defining the potencies of this series of benzoxaboroles in *T*. *brucei*.

Noticeably, among three aminomethylphenoxy isomers, there was a further variation in the impact of the knockdown on drug potency; the highest impact was observed with AN3057 (ΔEC_50_≈120), followed by AN3056 (ΔEC_50_≈7), and the least with AN3054 (ΔEC_50_≈4). This phenomenon suggests a differential contribution by Tb927.6.3050 to the anti-trypanosomal activities of aminomethylphenoxy bezoxaboroles with further chemical diversity. Additionally, we found in comparing the aminomethylphenoxy-carboxylate pair compounds that the methylamine containing compounds are more potent than the carboxylate counterparts, with ~8 fold difference between AN3057 and AN2861, ~3 fold between AN3054 and AN3330, and ~15 fold between AN3056 and AN3410.

### Tb927.6.5030 encodes a putative fatty aldehyde dehydrogenase (FALDH)

**Fig 3:**
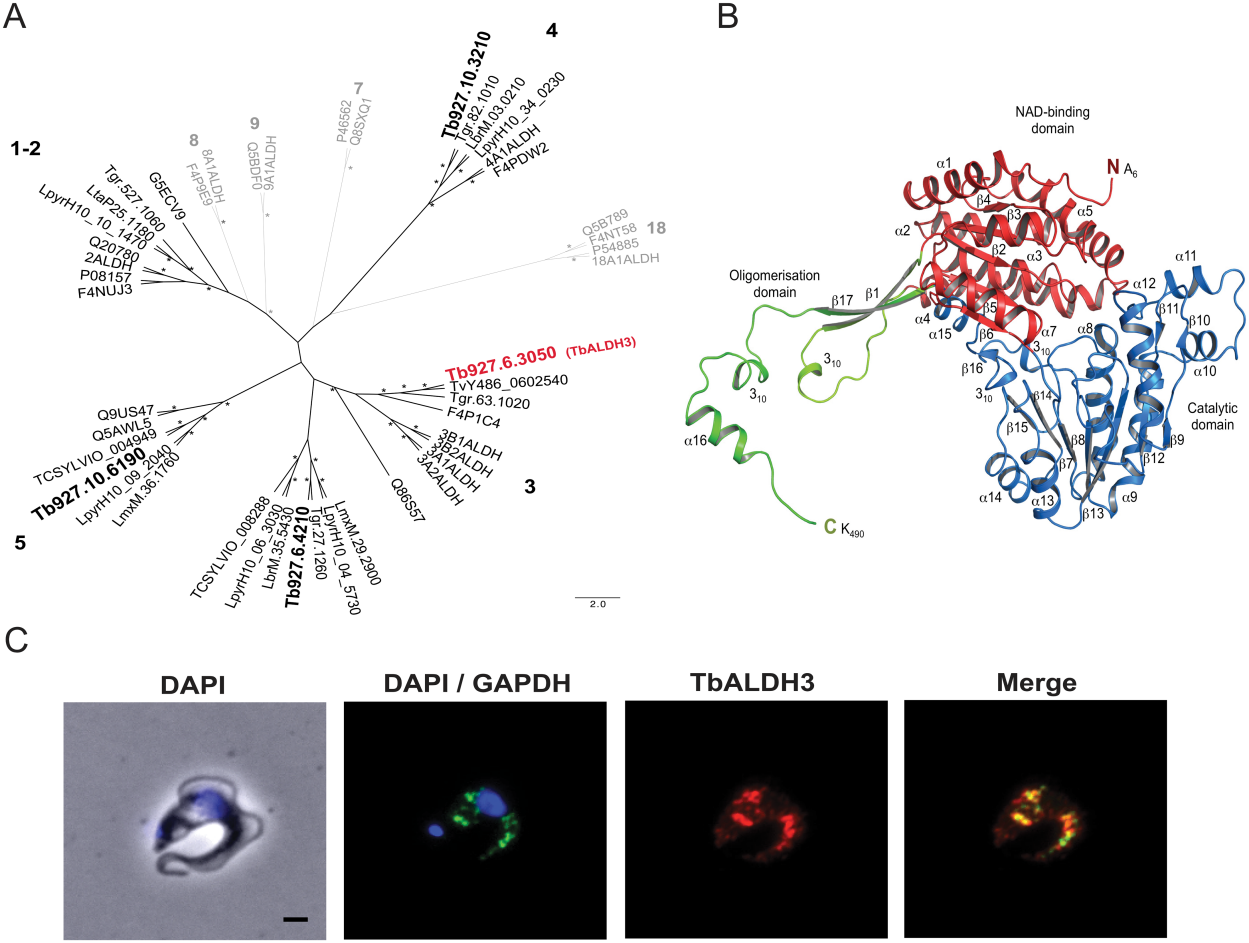
TbALDH3, encoded by Tb927.6.3050, is a member of FALDH subfamily. (**A**) The unrooted phylogenic tree was constructed based on the alignment of all ALDH orthologues in Trypanosomatida with the Opisthokont orthologues representing all the subfamilies of the ALDH superfamily categorized. Only representative orthologues are presented for simplicity. See S1 and S2 Figs for the complete data set. Each clade, in which the Trypanosomatida orthologues are identified with a Opisthokont subfamily, is assigned with the corresponding subfamily number, indicated in black; otherwise in grey. The family members in *T*. *brucei* are highlighted in a larger font with Tb927.6.3050 also in red. Strongly supported nodes (bootstrap proportion >70, Bayesian posterior probability >90) are indicated by a star. (**B**) The structure of TbALDH3 in cartoon representation. One subunit of the dimeric assembly is shown for clarity. Secondary structure elements are labeled and domains are indicated in color. Blue, catalytic domain; red, NAD-binding domain; green, oligomerisation domain. (**C**) The glycosomal localization of TbALDH3. The glycosomal localization is represented by the GAPDH signal, and the nucleus by DAPI stain. Scale bar = 1um.

Next, we took a comparative genomics approach to elaborate the function of Tb927.6.3050, which was previously uncharacterised [28]. We constructed a phylogenetic tree for all members of the ALDH superfamily currently known in humans and other opisthokonts (S1 Fig), and used this as reference to identify and categorise potential orthologues in the Trypanosomatida (S2 Fig). All candidates identified were categorized into five distinct clades based on the similarity of each towards the corresponding orthologue in the opisthokonts; a trypanosomatid-specific ALDH subfamily, represented by Tb927.6.4210 in *T*. *brucei*, emerged along with ALDH1/2, ALDH3, ALDH4 and ALDH5 subfamilies, indicating evolutionary functional divergence (Fig 3A). Importantly, Tb927.6.3050 falls into the clade comprising members of the ALDH3 subfamily that have been linked specifically to the metabolism of fatty acid and aromatic aldehydes [29], and thus is designated as TbALDH3 (Uniprot Q583M9).

Greater functional insight was attained from analyzing the TbALDH3 structure by X-ray crystallography. The structure of HsALDH3A1 [30] (PDB 1AD3) was used as the searching template to obtain the initial phases of the structure by molecular replacement. In the solved structure (PDB 5MYP), TbALDH3 appears as a dimer that consists of two monomeric polypeptides (S1 Table). As shown in Fig 3B, the monomer structure comprises 485 residues from A_6_ to K_490_, and adopts a canonical ALDH fold that is composed of an N-terminal NAD-binding domain, a catalytic domain and an oligomerization domain embracing the second subunit of the dimer. Several distinct features are also apparent in the structure, including an extended N-terminus with 15 residues preceding helix α1, a side-extrusion from the catalytic domain formed by helices α10 and α11 that are bridged by a flexible loop (G_297_-Q_303_), and an C-terminal extension adjacent to the dimerization domain (helix α16, Y_477_-L_489_). Interestingly, this C-terminal structural element strikingly resembles the ‘gatekeeper helix’ that is unique to FALDHs and is involved in regulating enzyme activity and substrate specificity [31].

Previously it has been noted that differential localizations of family members can contribute to the functional divergence in ALDHs [32–37]. By immunofluorescence, we found that TbALDH3 is predominantly co-localized with glyceraldehyde phosphate dehydrogenase (GAPDH). The latter is a distinctive marker for glycosomes, a specialised membrane-enclosed organelle in a few protozoan species including *T*. *brucei*, derived from peroxisomes and with essential metabolic functions (Fig 3C). This result is also supported by a recent quantitative glycosomal proteomics study that identified TbALDH3 with high confidence [38]. Overall this evidence strongly suggests that TbALDH3 functions as a glycosomal FALDH.

#### Aminomethylphenoxy benzoxaboroles can be metabolized by MAO-TbALDH3

Biogenic amines, exemplified by neurotransmitters, including serotonin and dopamine, are generally metabolized through two sequential enzymatic reactions; amine oxidases (AOs) such as monoamine oxidase (MAO) are responsible for an amine-to-aldehyde transition in the first step, while ALDHs in the second step further oxidize aldehyde metabolites into carboxylic acids [39, 40]. We hypothesized that a similar enzymatic cascade converts aminomethylphenoxy compounds into carboxylphenoxy metabolites, in which the first reaction is catalyzed by an AO, followed by a second oxidation by TbALDH3.

**Fig 4:**
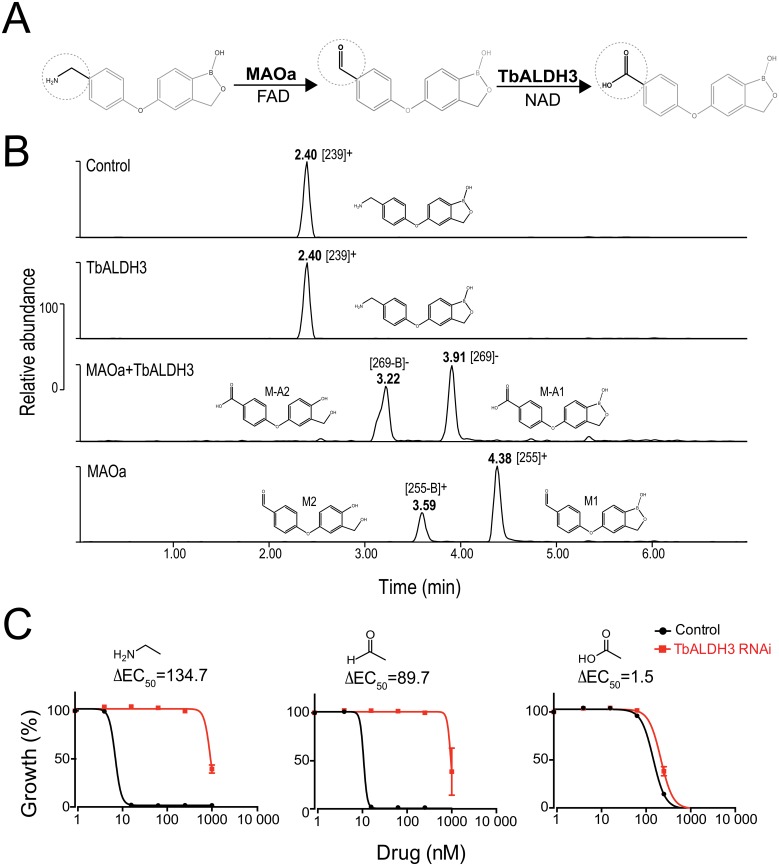
AN3057 is metabolized by MAO-TbALDH3. (**A**) Schematic view of the enzymatic reactions with MAOa-TbALDH3 in metabolizing AN3057. (**B**) HPLC-MS identification of AN3057-derived metabolites from the MAO-TbALDH3 pathway. HPLC peaks are indicated with corresponding retention time (RT) under acidic condition, m/z of ion precursors identified in MS, and proposed structural formula. See S2 Table for the RTs with acidic eluent or basic eluent; S3 Fig for the detailed MS data. (**C**) The potency (EC_50_) of AN3057 and derived metabolites in *T*. *brucei*. The change in EC_50_ upon TbALDH3 RNA*i* is presented for each molecule with the key moiety illustrated, i.e., methylamine, aldehyde, and carboxylic acid.

To test this hypothesis, we introduced AN3057 into an *in vitro* enzymatic cascade reaction, as shown in Fig 4A, and extracted the metabolites for HPLC-MS analysis. In the absence of MAOa (two upper panels in Fig 4B), there was no enzymatic conversion of AN3057 (CH_3_-NH_2_) by TbALDH3 as indicated by the unchanged retention time (RT) and the MS profile of the metabolite compared to the control. The MS m/z of 238 did differ from the theoretical m/z of 255.08, most likely due to the loss of a hydroxyl group (-OH) (S3 Fig). In contrast, two major metabolites, M-A1 and M-A2, were detected when both MAOa and TbALDH3 were present (middle panel in Fig 4B). The RTs of M-A1 and M-A2 were altered in an analogous manner upon changing the eluent pH from acidic to basic, suggesting that both metabolites contain an acid moiety (S2 Table). Additionally, the MS profile of M-A1 was identical to AN2861 (-CO_2_H) (S3 Fig), but different from M-A2, in which the boron atom was very likely lost as suggested by the changes in both elemental composition and isotope pattern, giving rise to a RT different from M-A1 (Fig 4B, S3 Fig). Interestingly, two metabolites (M1, M2) were generated with distinct MS profiles from either AN3057 (-CH_2_NH_2_) or AN2861 (-CO_2_H) when TbALDH3 was absent (lower panel in Fig 4B). Upon changing the eluent pH, there was no significant change in RT observed with M1 and M2, indicating that both of these metabolites are likely uncharged (S2 Table). Moreover, the difference in MS between M1 and M-A1 correlates well with the transition from aldehyde (-CHO) to carboxylic acid (-CO_2_H), as well as M2 and M-A2 (Fig 4B and S3 Fig), suggesting it is highly likely that M1 and M2 are the aldehyde precursors of the carboxylic acids M-A1 and M-A2 respectively. These data collectively support the notion that MAOa-TbALDH3 can metabolize the aminomethylphenoxy derivatives in a stepwise manner, i.e. the methylamine group in the structure is first converted by MAOa into an aldehyde, and then is further metabolized into a carboxylic acid by TbALDH3. It is also supported by the results with another isomer, AN3054, *albeit* with distinct kinetics that may reflect the variation in the efficiency of conversion (S4 Fig).

Next, we used the aldehyde and carboxylate metabolites generated as specific chemical probes to directly address if the trypanocidal activity of the aminomethylphenoxy benzoxaboroles is dependent on the function of this enzymatic pathway. We reasoned that if the enzymatic activity was essential for potency, silencing TbALDH3 by RNA*i* would lead to decreased potency for the parental compound (-CH_2_NH_2_) or the aldehyde metabolite (-CHO) but the carboxylate metabolite would remain unaffected. As shown in Fig 4C, substantial reductions in potency were observed for the parental compound (ΔEC_50_≈135) and for the aldehyde metabolite (ΔEC_50_≈90), in clear contrast to static potency for the carboxylate metabolite (ΔEC_50_≈1.5). Therefore, TbALDH3 action, as part of an AO-TbALDH3 enzymatic pathway, is indeed essential for trypanocidal activity of the aminomethylphenoxy benzoxaboroles.

### Molecular insights into the oxidation of the aldehyde metabolites by TbALDH3

**Fig 5:**
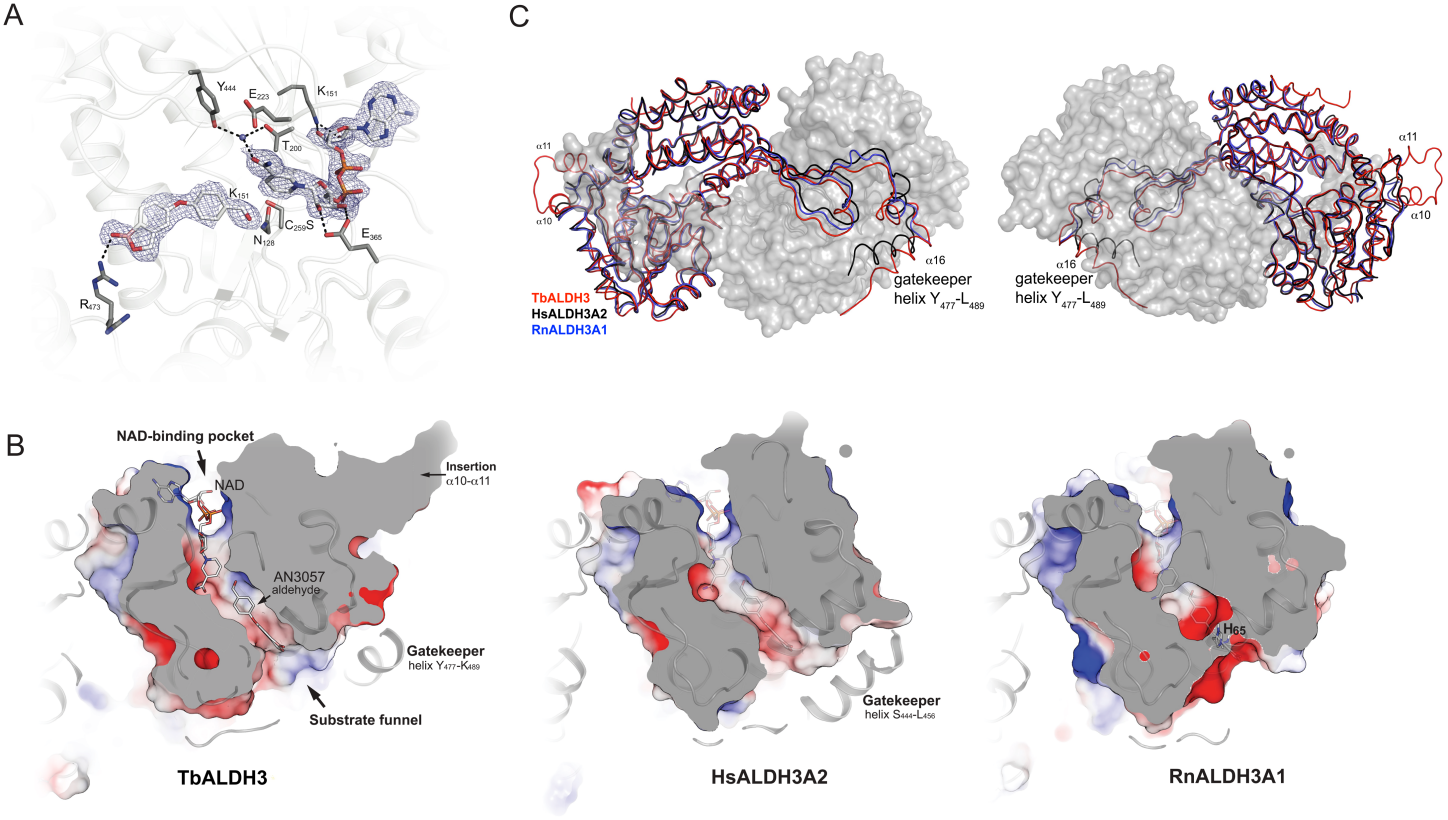
Structural insight into interactions between the aldehyde metabolites and TbALDH3. (**A**) TbALDH3 catalytic site. All key residues are in stick representation, while NAD and AN3057-aldehyde are shown with electron density (blue chicken wire), in which non-carbon atoms are marked in color, oxygen in red, nitrogen in blue, and phosphorus in orange. Potential hydrogen bonds are depicted as black dashed lines; selected water as blue spheres. (**B**) The comparison of substrate binding funnel between TbALDH3, HsALDH3A2 and RnALDH3A1. The cross-section structures are depicted from a top view with indicated features. NAD and AN3057-aldehyde are shown in solid stick representation with TbALDH3, while transparent superimposed to HsALDH3A2 and RnALDH3A1. (**C**) The structural superposition of TbALDH3 with mammalian homologues, RnALDH3A1 (Rat, blue) and HsALDH3A2 (Human, black). Two orientations are presented with indicated features. TbALDH3- RnALDH3A1 alignment: 1.4Å (rmsd); 445 (C_α_-atoms); 55 (Z-score); 47% (identity). TbALDH3-hALDH3A2: 1.8Å (rmsd); 454 (C_α_-atoms); 54 (Z-score); 47% (identity).

To understand the reaction catalyzed by TbALDH3 at molecular level, we obtained crystals of the aldehyde-NAD-TbALDH3 complex. A micro-reaction chamber (S5 Fig) was designed to serve as a physical barrier between the two reactions as well as to minimize the reverse reaction; meanwhile, a cysteine to serine mutation (C_259_S) was introduced in TbALDH3 to entrap the hyperactive aldehyde metabolites by abolishing the nucleophilic attack on the carbonyl carbon of the aldehyde. The resulting crystals are isomorphous to the apo-TbALDH3 crystals and the structure was solved at 2.5 Å (PDB 5NNO). As illustrated in Fig 5A, the NAD cofactor is located in an extended pocket rendered by a Rossmann fold [41]. There are two hydrogen bonds formed between E_365_ and the hydroxyl groups of the ribose of the nicotinamide, establishing the key interactions between NAD with TbALDH3 in addition to an H_2_O-bridge that connects the carboxylate group of the nicotinamide to Y_444_ and T_200_. Interestingly, similar topological arrangements have been proposed as part of the catalytic mechanism for ALDH. In this structure, the benzaldehyde benzoxaborole substrate exhibits distinctive electron density (S6 Fig), being nestled into the substrate funnel and adjacent to the nicotinamide (Fig 5B). Specifically, in the catalytic pocket, its aldehyde group directly contacts the catalytic residue (C_259_S), and also connects to N_128_, a residue whose counterparts in other ALDHs are involved in the catalytic activity by directing the carbonyl carbon towards the nucleophilic carbon on the nicotinamide [42, 43]. Noticeably, the position of the substrate is relatively flexible as indicated by the *B*-factor (S1 Table). There is an additional interaction between the substrate and TbALDH3, which is mediated by the hydrogen bond between the hydroxyl group of the hemiboronic ring in the substrate and a side-chain conformer of R_473_ in TbALDH3, proximal to the gatekeeper helix of the adjacent protomer (Fig 5B). In contrast, the boron atom remains in trigonal planar configuration, therefore capable of forming stable adducts with the potential targets, which suggests that the aldehyde metabolites serve as the substrates of TbALDH3 rather than the enzyme inhibitors as described in other cases [11, 13, 20].

The enzymatic specificity of TbALDH3 towards the aldehyde metabolite was further determined by superimposing the structure onto two other ALDH3 subfamily member structures, HsALDH3A2 and RnALDH3A1 (Fig 5C; see S7 Fig for the sequence alignment). The C-terminal ‘gatekeeper’ helix in HsALDH3A2, absent in RnALDH3A1, has been shown to be involved in defining its substrate specificity by partially occluding the substrate entrance from the ‘substrate funnel’. Interestingly, a similar structure is also defined in TbALDH3, although it is distant from the ‘substrate funnel’, probably imposing less restriction on the substrates (Fig 5B and 5C). This structural element in TbALDH3 appears to be held in position via the hydrogen bond between K_481_ and the backbone carbonyl of F_474_; highly conserved among human FALDHs (S8 Fig), and serving as a mechanical hinge to support the activity of FALDHs. A point mutation in HsALDH3A2 (K_447_E), equivalent to the K_481_E in TbALDH3, leads to a deficient enzymatic activity and is genetically linked to Sj*ö*gren–Larsson Syndrome [44]. More importantly, in the superimposition (Fig 5B), the benzaldehyde-benzoxaborole substrate can be accommodated by TbALDH3 and HsALDH3A2, but encounters steric clashes with RnALDH3A1, indicating that the unique topological arrangement in the substrate funnel of FALDHs such as HsALDH3A2 and TbALDH3 determines the substrate specificity besides the positioning of the C-terminal ‘gatekeeper’ helix. This also raises the possibility that the conversion of the aldehyde metabolites into carboxylic acids could also occur in the host, as the structural similarity suggests that human FALDHs, like HsALDH3A2, are likely to possess a substrate specificity similar to TbALDH3.

### Host-derived SSAO is the dominant oxidase mediating aminomethylphenoxy benzoxaborole metabolism

**Fig 6:**
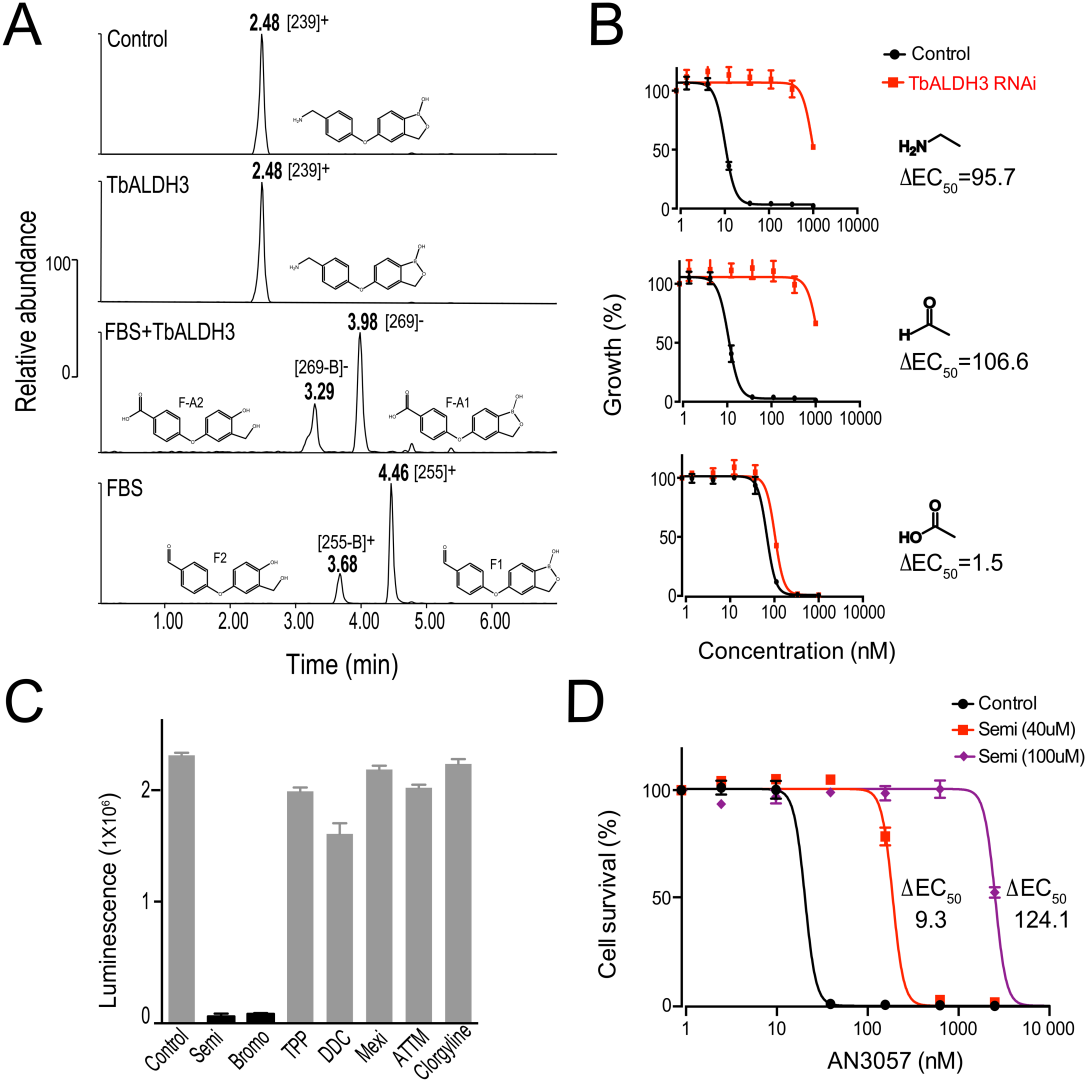
SSAO-TbALDH3, contributed by both host and parasites, metabolizes the aminomethyl-benzoxaboroles. (**A**) HPLC-MS identification of AN3057 and derived metabolites from the FBS-TbALDH3 pathway. HPLC peaks are indicated with corresponding retention time (RT), m/z of ion precursors identified in MS, and proposed structural formula. See S9 Fig for the detailed MS data. (**B**) The potency (EC_50_) of AN3057 and derived metabolites in *T*. *brucei*. The change in EC_50_ upon TbALDH3 RNA*i* is presented for each molecule with the key moiety illustrated, i.e., methylamine, aldehyde, and carboxylic acid. (**C**) SSAO contributes to the AO activity in FBS. FBS-derived AO activity is represented by the luminescence intensity from Luciferin, the generation of which is coupled to the aldehyde-carboxylic acid conversion. The chemical inhibitors are listed as follows, Semicarbazide (Semi), 2-Bromopropylamine hydrobromide (Bromo), Tetraphenylphosphonium (TPP), N,N-Diethyldithiocarbamate (DDC), Mexiletine (Mexi) and Ammonium tetrathiomolybdate (ATTM). (**D**) The potency (EC_50_) of AN3057 in *T*. *brucei* upon SSAO inhibition. Prior to the assay, the complete media were incubated with indicated concentrations of inhibitor for 4 hours.

The MAO-like enzymatic activity of the AO-TbALDH3 pathway remained undefined *in vivo*, considering the fact that neither genetic nor biochemical evidence supports the presence of such activity in *Trypanosoma* [45]. We postulated that the host provides this enzymatic activity, which is supported by documented AO activity in animal plasma [46–48]. Fetal bovine serum (FBS) is the only host-derived metabolically active component in our parasite culture system, and is the most likely source. To test this, we first analyzed the metabolism of AN3057 using an enzymatic cascade *in vitro*, composed of FBS and TbALDH3, by HPLC-MS (Fig 6A). The chemical transitions revealed resemble those through the MAOa-TbALDH3 pathway (Fig 4). No significant metabolic changes were detected with AN3057 in the absence of FBS, although the same loss of a hydroxyl group (-OH) occurred as observed previously with MAOa. Conversely, in the presence of both FBS and TbALDH3, two acidic metabolites (F-A1, F-A2) were identified, with F-A1 sharing the same MS profile with AN2861 and F-A2 subjected to the loss of boron (S9 Fig). Furthermore, when TbALDH3 was absent, AN3057 was metabolized via FBS into two metabolites (F1 and F2), both of which are suggested as similar benzaldehyde entities distinguished primarily by the boron in the structure (S9 Fig). In summary, AN3057 is converted by FBS first into the aldehyde metabolites (F1, F2), which are then further metabolized by TbALDH3 to the carboxylate metabolites (F-A1, F-A2). The metabolic pathway revealed here very likely mirrors the host-pathogen encounter in the host vascular system. To further validate this point, we examined the relative potencies of the metabolites, generated by the FBS-TbALDH3 *in vitro* cascade reactions, upon silencing TbALDH3 by RNA*i*. As shown in Fig 6B, with both the methylamine parent and aldehyde metabolites, the potency was significantly compromised following the RNA*i*. In contrast, no significant change in potency was observed with the carboxylate metabolites. Therefore, an FBS-TbALDH3 pathway can serve as a metabolic route through the host and the parasite for activating the aminomethylphenoxy benzoxaboroles.

Primary amine oxidases are generally classified into two major groups that are distinguished by the co-factor-dependent catalytic mechanism; one group is dependent on Flavin Adenine Dinucleotide (FAD) and represented by MAOa, while the second group, exemplified by Semicarbazide-Sensitive Amine Oxidase (SSAO), on copper-quinone [49, 50]. This biochemical feature differentiates the enzyme sensitivity to inhibitors that are specifically targeting the respective co-factors. Following this notion, we chose a fluorescence-reporter enzymatic assay to characterize the AO activity in FBS with a panel of specific AO inhibitors. As shown in Fig 6C, the enzymatic activity was exclusively sensitive to the SSAO inhibitors, particularly semicarbazide (Semi) and bromoethylamine (Bromo), and remained unaffected by MAO inhibitors, thus confirming that SSAO provides the predominant AO activity in FBS. More importantly, when we introduced semicarbazide into the trypanosome cultures, the potency of AN3057 was significantly compromised in a dose-dependent manner (Fig 6D), indicating the SSAO deamination activity derived from the host is critical for activating the aminomethylphenoxy benzoxaboroles as potent trypanocidals. Therefore, we identified the SSAO as the primary metabolic enzyme in the host intravascular system for metabolizing the chemical compounds containing primary amines via the oxidative deamination.

## Discussion

Benzoxaboroles have opened new pharmaceutical opportunities by combining unique chemical properties of the benzoxaborole core structure with a repertoire of structurally diverse substituents. This gives rise to a wide range of chemical entities of pharmaceutical interest that potentially act via diverse mechanisms in targeting various medical conditions. So far, the effort in understanding the MoA has been focused on identifying potential functional targets, with which the boron atom, as well as additional functional groups of the compounds, form stable interactions. For example, fungal or bacterial Leucyl-tRNA forms covalent bonds through the tRNA’s adenosine with the boron [16, 20]; CPSF3, an essential RNA metabolism factor in several protist parasites, is targeted primarily by the boron and the carboxylate group in AN3661 that form non-covalent bonds with the surrounding catalytic structures [11, 13]. However, a systemic understanding of the reciprocal actions between benzoxaboroles and targeted pathogens is lacking in general, particularly regarding the mechanisms underlying drug uptake and metabolism.

**Fig 7:**
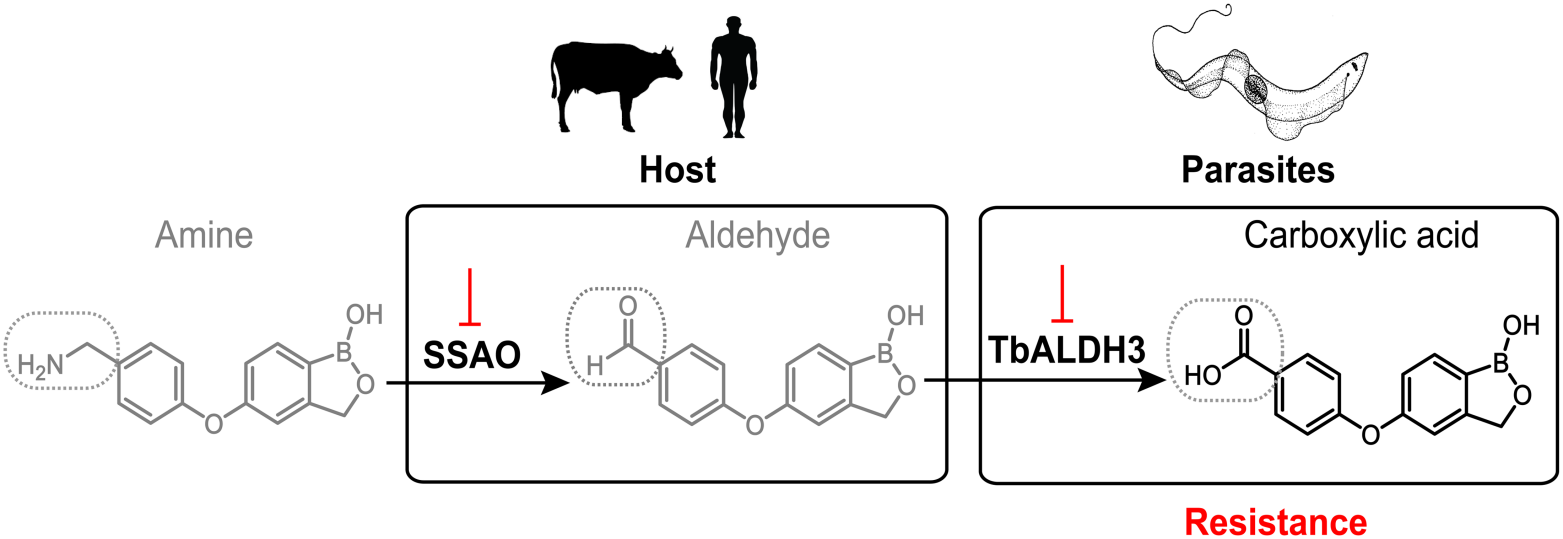
Model for aminomethylphenoxy benzoxaboroles metabolism *in vivo*. In the host, the aminomethylphenoxy benzoxaboroles are first oxidized by SSAO in the intravascular system. The resulting aldehyde metabolites are taken up by the parasites, and further oxidized into carboxylic acids that give rise to the final active trypanocides. The activation of the compounds requires both enzymatic activities in the host and parasites, with the loss of drug potency when either enzymatic activity is compromised. The key chemical moieties undergoing biochemical transitions through this process are highlighted with dotted boxes.

Here we exploited a genome-scale loss-of-function screen to unravel the complex interplay between structurally diverse benzoxaboroles and *T*. *brucei* as a pathogenic model. Part of the initial identifications suggested a specific SAR between the function conferred by an individual gene and aminomethylphenoxy derivatives, mediated by the methylamine moiety shared by the compounds. This gene encodes a protein, TbALDH3 that potentially functions as a trypanosomal FALDH based on our crystallographic and biochemical characterizations. More importantly, further evidence indicates that TbALDH3 contributes to an oxidative deamination enzymatic pathway that is initiated by a host AO activity and is required to achieve full potency of aminomethylphenoxy benzoxaboroles as trypanocides (Fig 7). Overall, this finding brings a drug metabolism perspective to our understanding of the MoA for benzoxaborole-based pharmaceutical entities, as well as highlights the possibility of similar metabolic pathways present in broader pathological contexts. Indeed, alternative routes could be constructed by divergent host AOs and pathogen-derived ALDHs, metabolizing structurally diverse compounds containing an amine moiety. Interestingly, a novel chemical route has been proposed for improving the potency of the antibiotics against Gram-negative pathogenic bacteria [51]. One of the key aspects was to introduce an amine group into the candidate molecules, resulting in effective drug accumulation in the pathogen, which implies more general applications for the drug metabolism mechanism uncovered here. We suggest that the potential exploitation of similar metabolic pathways on aminomethyl-containing drugs could be of significant value.

We also demonstrated that the carboxylate metabolites of the compounds exhibit the primary trypanocidal activity, although the same chemical entities, if applied directly, are less potent than the methylamine isomeric compounds and corresponding aldehyde metabolites. This lower potency with the carboxylate-containing benzoxaboroles is likely due to a less efficient drug uptake, as these compounds are negatively charged at neutral pH and hence might exhibit lower membrane permeability than uncharged aldehyde metabolites derived from methylamine pro-drugs. Noticeably, carboxylate benzoxaborole derivatives remain active as potent anti-parasitic candidates against *Plasmodium falciparum* and *Toxoplasma gondii* [11, 13]. Therefore, the drug metabolism mechanism revealed here can be exploited to improve the drug potency of these candidate compounds by replacing the carboxylate group with a methylamine. It would be also interesting to explore the uptake mechanism of these carboxylate benzoxaborole derivatives.

Our work indicates that the second oxidative reaction catalyzed by TbALDH3 likely occurs in glycosomes, therefore raising two possibilities of subcellular targeting mechanisms for the carboxylate benzoxaboroles as effective drugs, i.e. being either glycosomal or in alternative subcellular compartments. Nevertheless, the carboxylate derivatives such as AN2861 exhibit potent trypanocidal activities, independent of SSAO-TbALDH3 metabolic conversion. These data together suggest that the carboxylate benzoxaboroles are capable of overcoming the restriction, imposed by the negative charge of the compounds, on membrane permeability at both cellular and subcellular levels. This notion is also supported by our additional observation where there was no difference in the metabolomic profiles of parasites treated with AN3057 or with acoziborole (S3 Table), suggesting that similar ultimate impacts on the parasite targets are likely shared by structurally diverse benzoxaborole derivatives.

In addition to the oxidative deamination by AO-TbALDH3, we also observed an oxidative deboronation process, which has also been described elsewhere, especially in host plasma, for divergent classes of benzoxaboroles, where it accounts for a compromise in the efficacy of these compounds [52, 53]. Our observation raises a possibility that the oxidative enzymes in plasma such as AOs could be responsible for this oxidative deboronation reaction, therefore providing a possible route for improving the efficacy of benzoxaboroles in general. It also suggests the parasite culture system as a credible model in understanding drug metabolism through host and intravascular parasites. Meanwhile, the aldehyde metabolites derived from the amine-containing pro-drugs are relatively stable, allowing us to analyze them by MS and also to extract them for probing the activity of TbALDH3 directly in the parasites. It actually reflects the general feature of the ketone and aldehyde metabolites in various biological and pathological contexts such as diabetes and alcoholism[54].

Although further work is required to define the physiological functions of TbALDH3, it is reasonable to speculate that long chain fatty aldehydes are the most likely substrates for TbALDH3, based on phylogenetic and structural analyses, as well as a glycosomal localization of the enzyme. However, little is known in general about the role of glycosomal lipid metabolism in the adaptation of trypanosomes to the host environment, nor the potential roles of ALDHs in fatty acid metabolism in trypanosomes. Interestingly, adipose tissue has been recently demonstrated as a host reservoir for *T*. *brucei* [55], suggesting that further understanding of TbALDH3 and related FALDHs could provide unique insight into host-parasite interactions and compartmentalization of metabolic functions.

## Materials and methods

### RIT-seq and data analysis

Genetic determinants of drug resistance were identified using a *T*. *brucei* RNA*i* library screen as previously described [26]. Briefly, the library was grown under RNA*i*-inducing conditions (tetracycline, Tet at 1 mg.ml^-1^) 24 h prior to benzoxaborole drug-selection at 1.5~2xEC_90_. Through the screening, cultures were maintained and supplemented with fresh drug before extracting DNA from the drug-resistant population once it was established. RNA*i* target fragments within the population were amplified by PCR using LIB2f and LIB2r primers and the collective products were then subjected to high-throughput sequencing at BGI (The Beijing Genome Institute). The workflow for sequencing was as follows; the sequence libraries were constructed with the PCR products fragmented to ~300 bp and subjected to Illumina HiSeqTM4000. Reads were mapped to the *T*. *brucei* 927 reference genome (v6, tritrypdb.org) with Bowtie 2 [56] using the parameters: very-sensitive-local–phred33. Alignment files were manipulated with SAMtools [57] and a custom script [26], and data were further assessed using the Artemis genome browser [58].

### Drug potency (EC_50_)

EC_50_ was determined by exposing cells to the test compound at serial dilutions (2X or 4X as indicated in the corresponding data) with the highest concentration at 10 μM unless specified otherwise. The assays were conducted in 96-well plate format with three replicates for each sample and a non-treatment control in parallel [59]. The parasites were seeded at 1X10^4^ cells/ml and cultured for 72 h before adding resazurin. After 6h of incubation with resazurin, the fluorescence from resorufin was measured with a Gemini Fluorescent Plate reader (Molecular Devices) at an excitation wavelength of 530 nm, an emission wavelength of 585 nm and a filter cut-off of 570 nm. Each dataset shown is representative of three independent experiments (n = 3); in the case of RNA*i*, two independent RNA*i* clonal cultures were analysed.

### Plasmid construction and cell lines

For expressing recombinant proteins, the complete Tb427.06.3050 (TbALDH3) coding sequence was PCR amplified from genomic DNA and cloned into a modified pET27b vector (Novagen) creating pET27bTbALDH3. The plasmid produces TbALDH3 with a N-terminal hexa-histidine-tag followed by a tobacco etch virus (TEV) protease cleavage site. Catalytic cysteine to serine (C_259_S) mutation was generated by site directed mutagenesis using the Quikchange protocol (Stratagene). For RNA*i*, in brief, a gene-specific target sequence was selected through RNAit [60] and introduced into 2T1 cells using the pRPa^iSL^ vector [61]. Upon Tet-induction, dsRNAs were generated from the target sequence and the sequence-dependent RNA*i* was initiated in the transformed cells. For tagging the native locus, a sequence encoding a 6xMyc epitope was introduced into the native loci of Tb427.06.3050 using the pNATx^TAG^ vector, resulting in the strain expressing TbALDH3 with a C-terminal epitope. The integrity of all constructs was verified by sequencing. See S4 Table for the details of constructs and the primers for cloning. All the transformed *T*. *brucei* bloodstream strains were established and maintained as previously described [61].

### Phylogenetic analysis

Sequences of putative aldehyde dehydrogenases were retrieved from a BLASTp [62] search using *Homo sapiens* ALDH proteins as query against the genomes of the selected Opisthokont and Trypanosomatida species (BLOSUM62, Existence: 11 Extension: 1 Conditional compositional score matrix adjustment). The subsequent hits were analysed and outliers excluded from the phylogenetic assembly based on protein alignment generated using EMB-EBI Clustal Omega.

The curated list of proteins was again analysed with Clustal Omega and manually edited to remove the poorly aligned regions of the C and N terminus. Phylogenetic reconstructions were generated using PhyML [63] and MrBayes [64] using the default parameters with a 1000 bootstrapping and 800000 generations, respectively. Resulting trees were visualized using FigTree (http://tree.bio.ed.ac.uk/software/figtree/). At least two members of each clade in the individual Opisthokont tree were selected and aligned with a representative of the *Leishmania sp*., the *Leptomonas sp*., the African and the American *Trypanosoma sp*. proteins for each clade to reconstruct the phylogeny of aldehyde dehydrogenases. No ALDH6, ALDH16 or ALDH18 orthologues were identified in Trypanosomatida and their branches have been omitted from the Trypanosomatida-Ophistokont tree for simplification; ALDH families 7, 8 and 9 are shown in grey for the same reason. See S5 Table for the details of the species and the sequences with accession numbers.

### Recombinant protein expression and purification

Recombinant TbALDH3 protein was obtained by cultivating freshly transformed *E*. *coli* BL21 (DE3) Rosetta Gami 2 (Novagen) in a starter culture of Luria-Bertani (LB) medium in 20 mL, supplemented with 50 μg/mL of kanamycin and 15 μg/mL of chloramphenicol. 5 mL of the starter culture was then inoculated with the selective LB culture (4 L) with 1 mM magnesium chloride and 0.5 mM calcium chloride, grown at 37°C in 5 L Erlenmeyer flasks. OD_600_ was measured until it reached 0.6–0.8, at which point expression was induced with 1 mM IPTG (isopropyl-β-D-thiogalactopyranoside) and the culture incubated overnight at 25°C. The cells were harvested by centrifugation at 5000 g for 30 minutes at 4°C and were resuspended in buffer A (50 mM sodium phosphate pH 7.8, 300 mM sodium chloride, 10% glycerol) along with a protease inhibitor tablet (Roche). The cell-buffer A was passed through a pressure cell homogeniser (SPCH-10, Stansted), supplemented with 25 mM imidazole and passed through a 0.45 μm filter. A 1 mL CV (column volume) His-Trap HP column (GE Healthcare) charged with Ni^2+^ was calibrated by 4 CV washes with buffer B (50 mM sodium phosphate pH 7.8, 25 mM imidazole, 300 mM sodium chloride, 10% glycerol) after which the sample was loaded and fractions collected over a linear increase in imidazole concentration (25–250 mM over 18 CV). Fractions containing the eluted recombinant TbALDH3 protein were combined and concentrated in a spin concentrator (Millipore) exchanging the buffer to buffer A and the sample incubated overnight with His-tagged TEV protease (molar ratio of 1:20 TEV:recombinant protein). The sample was passed through a His-Trap HP column previously equilibrated with a buffer A, to separate cleaved enzyme from non-cleaved protein and the TEV protease itself. Fractions containing TbALDH3 were concentrated and chromatographed through a 24 mL CV Superdex75 GL (HR 10/300, GE Healthcare) size exclusion chromatography column equilibrated with buffer A. The size exclusion chromatography columns had been calibrated with molecular mass standards (thyroglobulin, 670 kDa; gamma-globulin, 158 kDa; serum albumin, 67 kDa; ovalbumin; 44 kDa, myoglobin, 17 kDa; vitamin B12, 1 kDa). Fractions containing purified TbALDH3 were combined, concentrated and used in crystallisation trials. The yield of TbALDH3 was estimated on the basis of theoretical molar extinction coefficients at 280 nm of 42860 M^-1^ cm^-1^, respectively, calculated using the software VectorNTI (Invitrogen).

### Crystallography

TbALDH3 crystals, maximum dimension 0.5 mm, were obtained in five days by sitting-drop vapour diffusion at 20°C in 3 μL drops in the ratio of 1:1 protein solution (20 mg/mL TbALDH3 in 50 mM sodium phosphate pH7.8, 300 mM sodium chloride, 10% glycerol) to reservoir condition (100 mM Bis-Tris pH 5.5, 0.2 M LiSO4, 25% PEG3350). For co-crystallisation with the aldehyde form of AN3057 the purified catalytically inactive TbALDH3C259S was concentrated to 27 mg/ml in buffer A supplemented with 2 mM NAD^+^, 2 mM MgCl_2_ and dialysed (3500 MWCO) for 14 h at RT against the same buffer containing 10 μM MAO-A (Sigma Aldrich) and 1 mM AN3057 using a homemade microdialysis device, then used in the same crystallization setup as before. TbALDH3 crystals, though of good appearance, displayed diffraction indicating the presence of multiple crystalline components. This problem was more dominant in the co-crystals and numerous crystals had to be screened to identify a crystal with twinning-properties down to acceptable levels.

Crystals were harvested using a glycerol or LV-oil (MiTeGen) cryoprotectant, flash frozen in liquid nitrogen and characterised in-house with a Rigaku MicroMax 007HF generator equipped with VariMax VHF optic, and a Saturn944 HG+ CCD detector. The data were indexed and merged using XDS [65] and SCALA [66], respectively. The crystals displayed the space group P1211. The crystal structure of the wild-type apoprotein was solved to 1.95 Å using the MR protocol of Auto-rickshaw [67] using a 432 residues search model built using PHYRE2 [68]. Density modification was performed using PIRATE [69], as implemented in the CCP4 suite of programs [70], and the model was extended to 920 residues with ARP/wARP [71]. Refinement with Refmac5 [72] resulted in R_work_ and R_free_ of 0.19 and 0.24, respectively. This model was inspected, along with electron density and difference density maps, adjusted and extended to 973 residues using COOT [73]. Translation/Libration/Screw (TLS) refinement [74] in Refmac5 with multiple rounds of electron and difference density map inspection, model manipulation and the inclusion of water molecules, dual conformers and glycerol completed the refinement. The apoprotein structure served as a search model to phase native data collected from the isomorphous co-crystals to 2.5 Å by molecular replacement with PHASER. Rounds of model adjustment using COOT, interspersed with rounds of Refmac5 intensity based twin-refinement calculations, the addition and refinement of AN3057 aldehyde, NAD, water molecules, and inclusion of multiple conformers completed the refinement. Geometrical restraints for the AN3057 aldehyde intermediate were generated using the Grade Web Server (http://grade.globalphasing.org).

MOLPROBITY [75] and COOT were used to monitor model geometry during TbALDH3 refinement. Figures were prepared using PyMOL (Schrödinger LLC). The DALI server was used to search the PDB for structural homologues and structural superpositions were performed using DALILITE [76]. Multiple sequence alignments were calculated using CLUSTALW2 [77] and edited using ALINE [78]. Crystallographic statistics are presented in S1 Table.

### Chemicals and antibodies

AN3057, (4-(1-hydroxy-1,3-dihydrobenzo[c][1,2]oxaborol-5-yloxy)phenyl) methanaminium chloride, was synthesized as described [79], to a 4.78 mmol solution of 1.2 g of 4-(1-hydroxy-1,3-dihydrobenzo[c][1,2]oxaborol-5-yloxy) benzonitrile in EtOH (150 mL) under N_2_ was added Pd/C (10 wt.%, 0.178 g). The reaction mixture was hydrogenated for 26.5 hours using a H_2_ balloon at room temperature with stirring. The mixture was filtered, rotary evaporated and purified by silica gel column eluted with MeOH containing 0.6% volume NH_4_OH (3 mL 28–30% NH_4_OH to 500 mL MeOH). The white solid obtained was dissolved in water (80 mL) and 6N HCl (2 mL) was added, filtered and the filtrate was lyophilized to give the desired salt (4-(1-hydroxy-1,3-dihydrobenzo[c][1,2]oxaborol-5-yloxy)phenyl)methanaminium chloride as white solid (0.93 g, 3.19 mmol, yield 66.7%). M.p. > 250°C. ^1^H-NMR (DMSO-d_6_, 300 MHz): δ 9.18 (s, 1H), 8.43 (br. s, 3H), 7.74 (d, *J* = 8.1 Hz, 2H), 7.52 (d, *J* = 8.7 Hz, 2H), 7.08 (d, *J* = 8.7 Hz, 1H), 6.98–6.94 (m, 2H), 4.91 (s, 2H) and 3.99 (br. q, *J* = 4.8 Hz, 2H) ppm. Purity (HPLC): 94.9% at 254 nm. MS: m/z = 256 (M+1, ESI+) and m/z = 255 (M-, ESI-). The method is summarized in S10 Fig. AN2861 was described by Yong-Kang *et al*. [80], while AN3054, AN3056, AN3330, AN3410 were described by Akama *et al*. [81]. The chemicals purchased from Sigma are as follows, Tetracycline hydrochloride (T7660), Clorgyline (M3778), Semicarbazide (S2201), Tetraphenylphosphonium chloride (218790), Sodium diethyldithiocarbamate trihydrate (D3506), 2-Bromoethylamine hydrobromide (B65705), Mexiletine hydrochloride (M2727), Ammonium tetrathiomolybdate (323446), Resazurin (R7017), anti-Myc (commercial).

### HPLC-MS

Samples were analyzed on a Waters Xevo Q-TOF Mass Spectrometer coupled to a Waters Acquity UPLC system using a Waters BEH C18 column 50x2.1mm 1.6 μm. Analysis was done in either acidic (A: H_2_O + 0.1% formic acid, B: ACN + 0.1% formic acid) or basic eluents (A: H_2_O + 0.1% NH3 solution, B: ACN + 0.1% NH3 solution). The same gradient was used with both types of eluent: 98% A for 0.5 min then linear gradient to 65% A (3.5 min) then to 5% A in 1 min, gradient held for 1 min before a step change back to the to the starting conditions and an equilibration time of 1 min (total run time 7 min).

The mass spectrometer was operated in both positive (Capillary Voltage 2.3 kV) and negative mode (Capillary Voltage 1.5 kV). Source temperature and desolvation gas temperatures were constant at 120°C and 500°C respectively. In MS operation, spectra were acquired every 0.2 s over a 50–1000 amu range.

### Amine oxidase activity assay

MAO-Glo assay (Promega, V1401) was applied to determine MAO and SSAO activities respectively according to manufacturer’s instructions. Recombinant human MAO A (Sigma, M7316) was included as the positive control, and the assay was conducted with following the optimized protocol [82].

### Immunofluorescence

*Tb*ALDH3 localization was determined using a native C-terminal 6xMyc epitope fusion. Samples were prepared as previously described. Antibodies were used at the following dilutions: rabbit anti-myc epitope IgG (Santa Cruz Biotechnology Inc.) at 1:500, and mouse anti-TbGAPDH (a kind gift from M.A.J. Ferguson, Dundee) at 1:1000. Secondary antibodies (Life Technologies) were Alexa Fluor 568 conjugated anti-rabbit IgG (1:1000) and Alexa Fluor 488 conjugated anti-mouse (1:1000). Coverslips were mounted using *Prolong Gold* mounting medium supplemented with 4’,6-diamidino-2-phenylindole (DAPI) (Life Technologies). The cells were examined on a Zeiss Axiovert 200M microscope and images captured with a AxioCam MRm camera. Digital Images were captured and processed using Zen Pro software (Zeiss) and Adobe Photoshop CS3 (Adobe Systems Inc.).

### Metabolite extraction and analysis

Metabolite extractions were performed as previously described [83]. Briefly, bloodstream form cells were treated with 5xEC_50_ of drug in HMI-9 for the time required for growth to be inhibited (6 hours for AN5568, 8 hours for AN3057). 1 x 10^8^ cells were centrifuged and extracted for one hour, shaking in 200 μL UPLC grade chloroform:methanol:water (1:3:1) on ice. Samples were centrifuged and stored at -80°C before being run on a QExactive mass spectrometer (Thermo) after separation on a zic-pHILIC column (Sequant) according to previously published methods. Raw data were filtered and aligned and annotated using the Orbiwarp algorithm in PiMP (http://polyomics.mvls.gla.ac.uk/) was used to match masses and retention times to authentic standards, to provide annotations and to perform statistical analyses.

## Supporting information

S1 Fig Phylogenetic analysis of ALDH family in Opisthokont. Each clade is labelled with the corresponding subfamily number. All human orthologues are highlighted in bold. The nodes with significant bootstrap value (>0.6) and Bayesian posterior probability (>90%) are indicated with stars.
(PDF)

S2 Fig Phylogenetic analysis of ALDH family in Trypanosomatida. Each clade is assigned in reference to the Opisthokont subfamilies, and labelled with the corresponding subfamily number identified with. The nodes with significant bootstrap value (>0.6) and Bayesian posterior probability (>90%) are indicated with stars. The family members in *T*. *brucei* are highlighted in a larger font with Tb927.6.3050 also in red.
(PDF)

S3 Fig MS spectrums of AN3057, AN2861 and AN3057-derived metabolites from MAOa-TbALDH3. (**A**) MS spectrums of the ion precursors from either AN3057 or the metabolite from AN3057+TbALDH3. The [M+H]_+_ ion at *m/z* 239.0 was identified with both, indicating no change in structure. (**B**) MS spectrums of the ions precursors from either AN2861 or the metabolites from AN3057+MAOa+TbALDH3. The [M-H]_-_ ion at *m/z* 269.0 was identified with both AN2861 and M-A1 metabolite, indicating an identical structure, which is consistent with detecting the [M+H]_+_ ion at *m/z* 271.0 from M-A1. The [M-H]_-_ ion at *m/z* 259.0 was identified with M-A2 metabolite, resulted from the loss of boron, consistent with detecting the [M-H2O]_+_ ion at *m/z* 243.0 from M-A2. (**C**) MS spectrums of the ions precursors from the metabolites derived from AN3057+MAOa. The [M+H]_+_ ion at *m/z* 255.0 was identified with M1 metabolite, suggesting a methylamine- aldehyde conversion occurred. The [M+H]_+_ ion at *m/z* 245.0 was identified from M2, indicating the loss of boron, consistent with detecting the [M-H]_-_ ion at *m/z* 243.0.
(PDF)

S4 Fig HPLC-MS analysis of AN3054-derived metabolites from MAOa-TbALDH3. (**A**) HPLC peaks are indicated with corresponding retention time (RT), m/z of ion precursors identified in MS, and proposed structural formula. See S5 Fig for the detailed MS data. (**B**) MS spectrums of the ion precursors from either AN3054 or the metabolite from AN3054+TbALDH3. The [M+H]_+_ ion at *m/z* 239.0 was identified with both, indicating no change in structure. (**C**) MS spectrums of the ions precursors from either AN3330 or the metabolites from AN3054+MAOa+TbALDH3. The [M-H]_-_ ion at *m/z* 269.0 was identified with both AN3330 and M-A1 metabolite, indicating an identical structure, which is consistent with detecting the [M+H]_+_ ion at *m/z* 271.0 from M-A1. The [M-H]_-_ ion at *m/z* 259.0 was identified with M-A2 metabolite, resulted from the loss of boron, consistent with detecting the [M-H2O]_+_ ion at *m/z* 243.0 from M-A2. (**d**) MS spectrums of the ions precursors from the metabolites derived from AN3054+MAOa. The [M+H]_+_ ion at *m/z* 255.0 was identified with M1 metabolite, suggesting a conversion from methylamine-aldehyde occurred. The [M+H]_+_ ion at *m/z* 245.0 was identified from M2, indicating the loss of boron, consistent with detecting the [M-H]_-_ ion at *m/z* 243.0.
(PDF)

S5 Fig Microdialysis device for binding the enzymatically generated TbALDH3 substrate. A 3.5 kDa dialysis membrane separates the two reservoirs allowing the diffusion of the benzaldehyde-benzoxaborole intermediate once formed by MaoA (lower reservoir) and subsequent binding by the catalytically inactive TbAlDH3 C259S mutant.
(PDF)

S6 Fig *Fo–Fc* omit map contoured at 2.0 σ (green chicken wire) and interactions for NAD+ and the AN3057-aldehyde ligand bound to TbALDH3. All key residues are in stick representation, in which non-carbon atoms are marked in color, oxygen in red, nitrogen in blue, and phosphorus in orange. Potential hydrogen bonds are depicted as black dashed lines;; selected water molecules as blue spheres.
(PDF)

S7 Fig Multiple sequence alignment of selected ALDHs. from *T*. *brucei* and *H*. *sapiens* (*Hs*ALDH3A1, P30838;; *Hs*ALDH3A2, P51648;; *Hs*ALDH3B1, P43353;; *Hs*ALDH3B2, P48448). Secondary structure elements are indicated for *Tb*ALDH3A2 (colored by domain: blue, catalytic domain; red, NAD-binding domain; green, oligomerisation domain) and *Hs*ALDH3A2 (colored in grey, from pdb 4QGK). The predicted C-terminal transmembrane segment in *Hs*ALDH3A2 is indicated and the prenylation/palmitoylation sites in *Hs*ALDH3B1 (Cys463 and Cys465) are highlighted in yellow. Note that the respective region in *Tb*ALDH3A2 does not to exhibit predicted transmembrane propensities and is lacking cysteine residues for lipidation.
(PDF)

S8 Fig Position of the gatekeeper helix. Structural superposition in cartoon representation of TbALDH3 (red) with HsALDH3A2 (grey) focussing on the gatekeeper helix and the conserved hydrogen bonding interaction (dashed line) between K481 and the backbone carbonyl of F474.
(PDF)

S9 Fig MS spectrums of AN3057, AN2861 and AN3057-derived metabolites from FBS-TbALDH3. (**A**) MS spectrums of the ion precursors from either AN3057 or the metabolite from AN3057+TbALDH3. The [M+H]_+_ ion at *m/z* 239.0 was identified with both, indicating no change in structure. (**B**) MS spectrums of the ions precursors from either AN2861 or the metabolites from AN3057+FBS+TbALDH3. The [M-H]_-_ ion at *m/z* 269.0 was identified with both AN2861 and F-A1 metabolite, indicating an identical structure, which is consistent with detecting the [M+H]_+_ ion at *m/z* 271.0 from F-A1. The [M-H]_-_ ion at *m/z* 259.0 was identified with F-A2 metabolite, resulted from the loss of boron, consistent with detecting the [M-H2O]_+_ ion at *m/z* 243.0 from F-A2. (**C**) MS spectrums of the ions precursors from the metabolites derived from AN3057+FBS. The [M+H]_+_ ion at *m/z* 255.0 was identified with F1 metabolite, suggesting a conversion from methylamine-aldehyde occurred. The [M+H]_+_ ion at *m/z* 245.0 was identified from F2, indicating the loss of boron, consistent with detecting the [M-H]_-_ ion at *m/z* 243.0.
(PDF)

S10 Fig Synthesis of (4-(1-hydroxy-1,3-dihydrobenzo[c][1,2]oxaborol-5-yloxy)phenyl)methanaminium chloride (AN3057). To the solution of 4-(1-hydroxy-1,3-dihydrobenzo[c][1,2]oxaborol-5-yloxy)benzonitrile (1.2 g, 4.78 mmol) in EtOH (150 mL) under N2 was added Pd/C (10 wt.%, 0.178 g). The reaction mixture was hydrogenated for 26.5 h using a H2 balloon at room temperature with stirring. The mixture was filtered, rotary evaporated and purified by silica gel column eluted with MeOH containing 0.6%volume NH4OH (3 mL 28–30% NH4OH to 500 mL MeOH). The white solid obtained was dissolved in water (80 mL) and 6N HCl (2 mL) was added, filtered and the filtrate was lyophilized to give the desired salt (4-(1-hydroxy-1,3-dihydrobenzo[c][1,2]oxaborol-5-yloxy)phenyl)methanaminium chloride as white solid (0.93 g, 3.19 mmol, yield 66.7%). M.p. > 250°C. ^1^H-NMR (DMSO-d6, 300 MHz): δ 9.18 (s, 1H), 8.43 (br. s, 3H), 7.74 (d, J = 8.1 Hz, 2H), 7.52 (d, J = 8.7 Hz, 2H), 7.08 (d, J = 8.7 Hz, 1H), 6.98–6.94 (m, 2H), 4.91 (s, 2H) and 3.99 (br. q, J = 4.8 Hz, 2H) ppm. Purity (HPLC): 94.9% at 254 nm. MS: m/z = 256 (M+1, ESI+) and m/z = 255 (M-, ESI-).
(PDF)

S1 Table TbALDH3 crystallography and refinement statistics. (PDF)

S2 Table HPLC-MS analysis of AN3057-derived metabolites from MAOa- TbALDH3. (PDF)

S3 Table Metabolism profiles of *T*. *brucei* with the treatment of different benzoxaboroles. (PDF)

S4 Table Primers and constructs used in the work. (PDF)

S5 Table Species, strains and protein acessions included in the phylogenetic analyses. (PDF)
