# Supplementary figures and images for: Host-parasite co-metabolic activation of antitrypanosomal aminomethyl-benzoxaboroles
Source: PLoS Pathog. 2018 Feb 9;14(2):e1006850. doi: 10.1371/journal.ppat.1006850 (PMC5823473; doi:10.1371/journal.ppat.1006850)

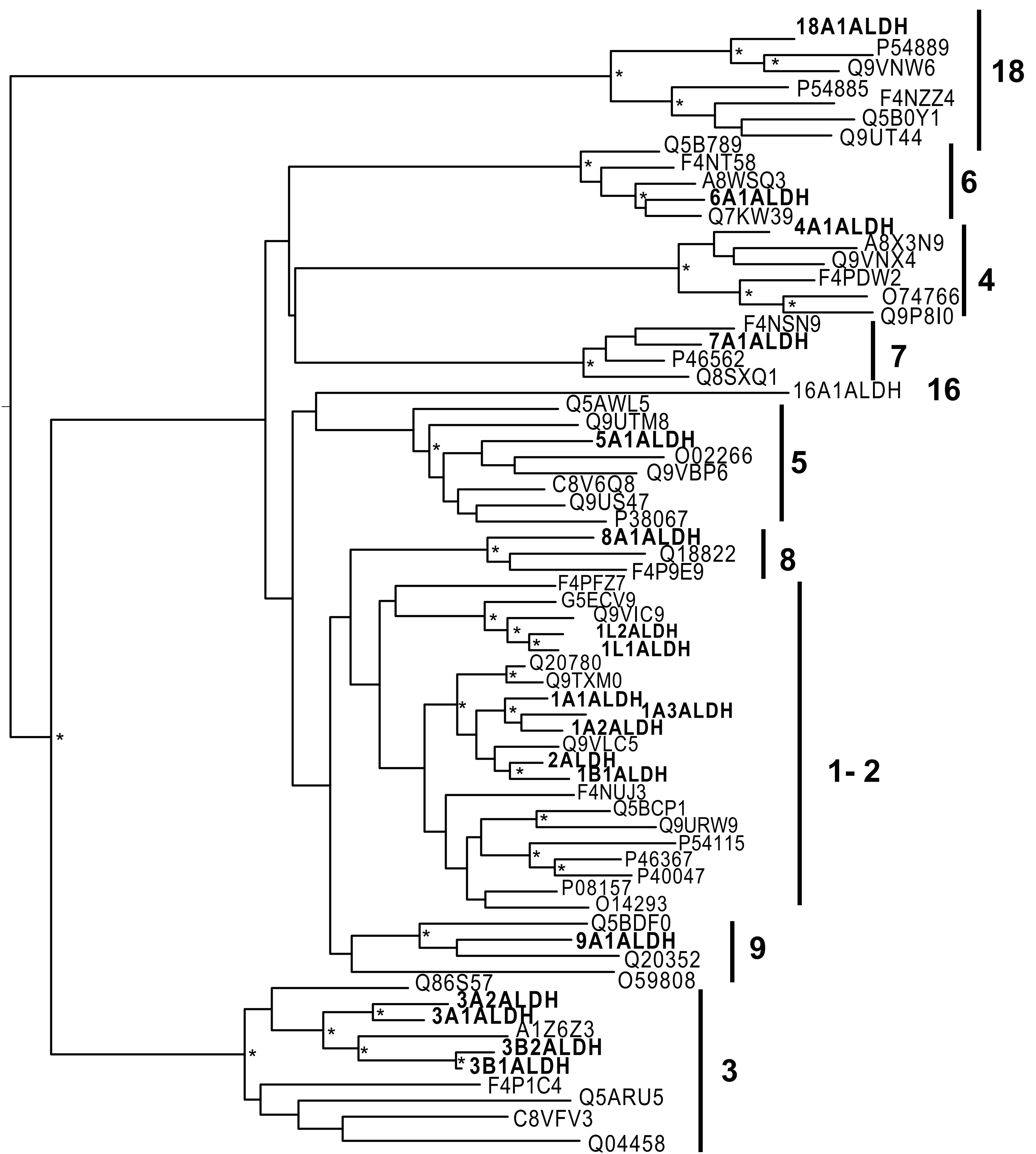

0.5

Supplement: S1 Fig — Each clade is labelled with the corresponding subfamily number. All human orthologues are highlighted in bold. The nodes with significant bootstrap value (>0.6) and Bayesian posterior probability (>90%) are indicated with stars. (PDF) [file ppat.1006850.s001.pdf]

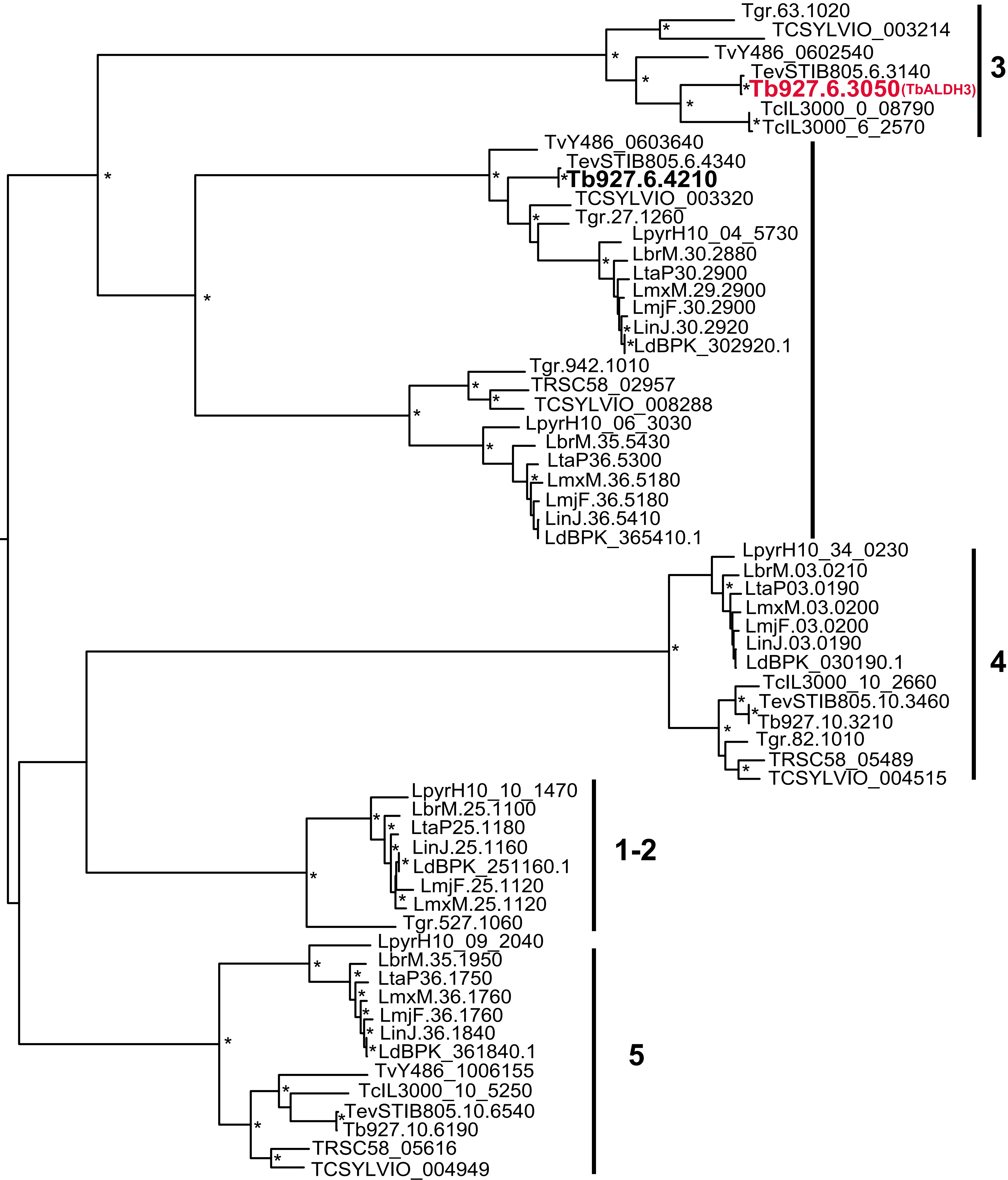

Supplement: S2 Fig — Each clade is assigned in reference to the Opisthokont subfamilies, and labelled with the corresponding subfamily number identified with. The nodes with significant bootstrap value (>0.6) and Bayesian posterior probability (>90%) are indicated with stars. The family members in T. brucei are highlighted in a larger font with Tb927.6.3050 also in red. (PDF) [file ppat.1006850.s002.pdf]

**A**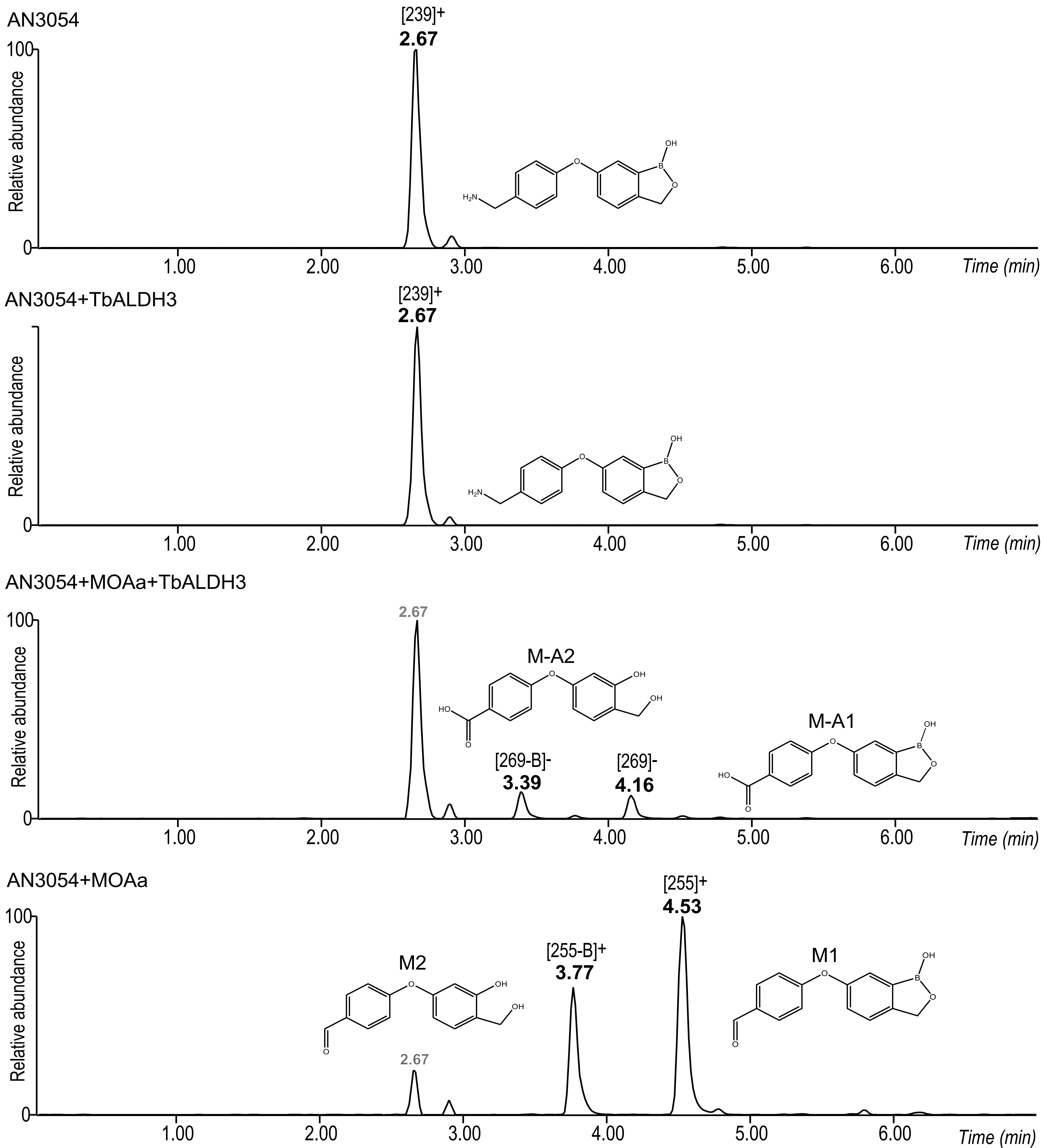**B**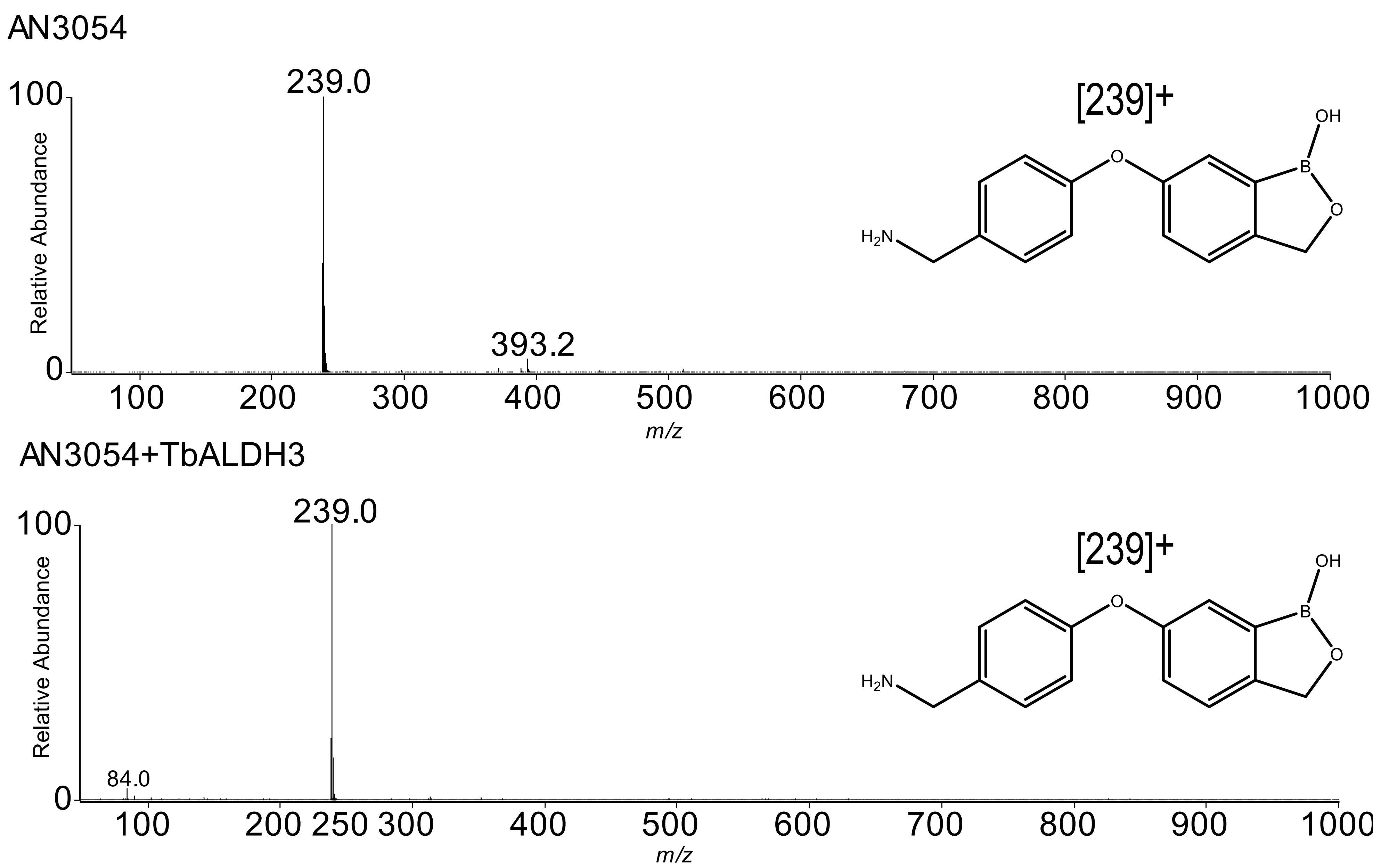**C**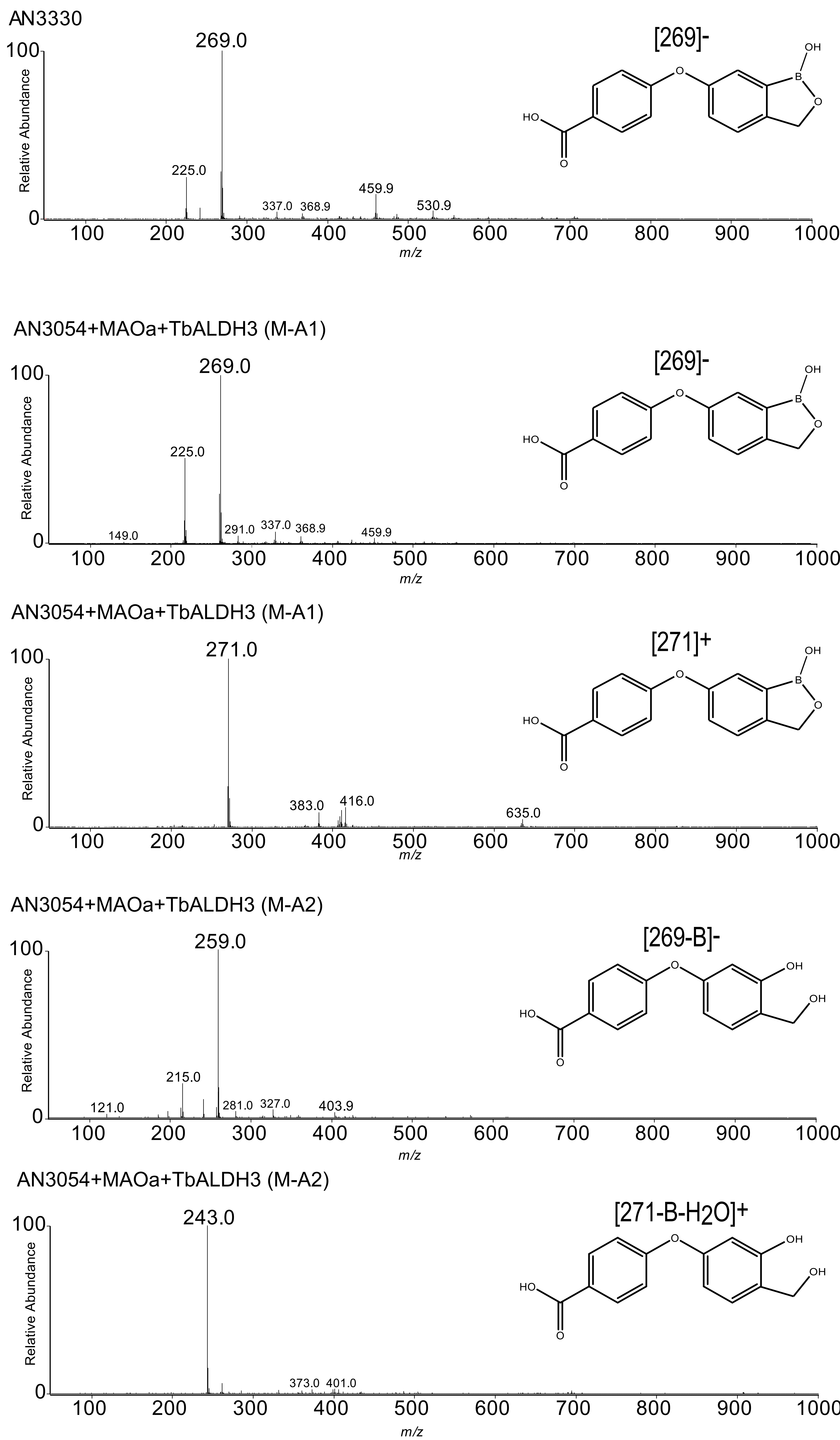**D**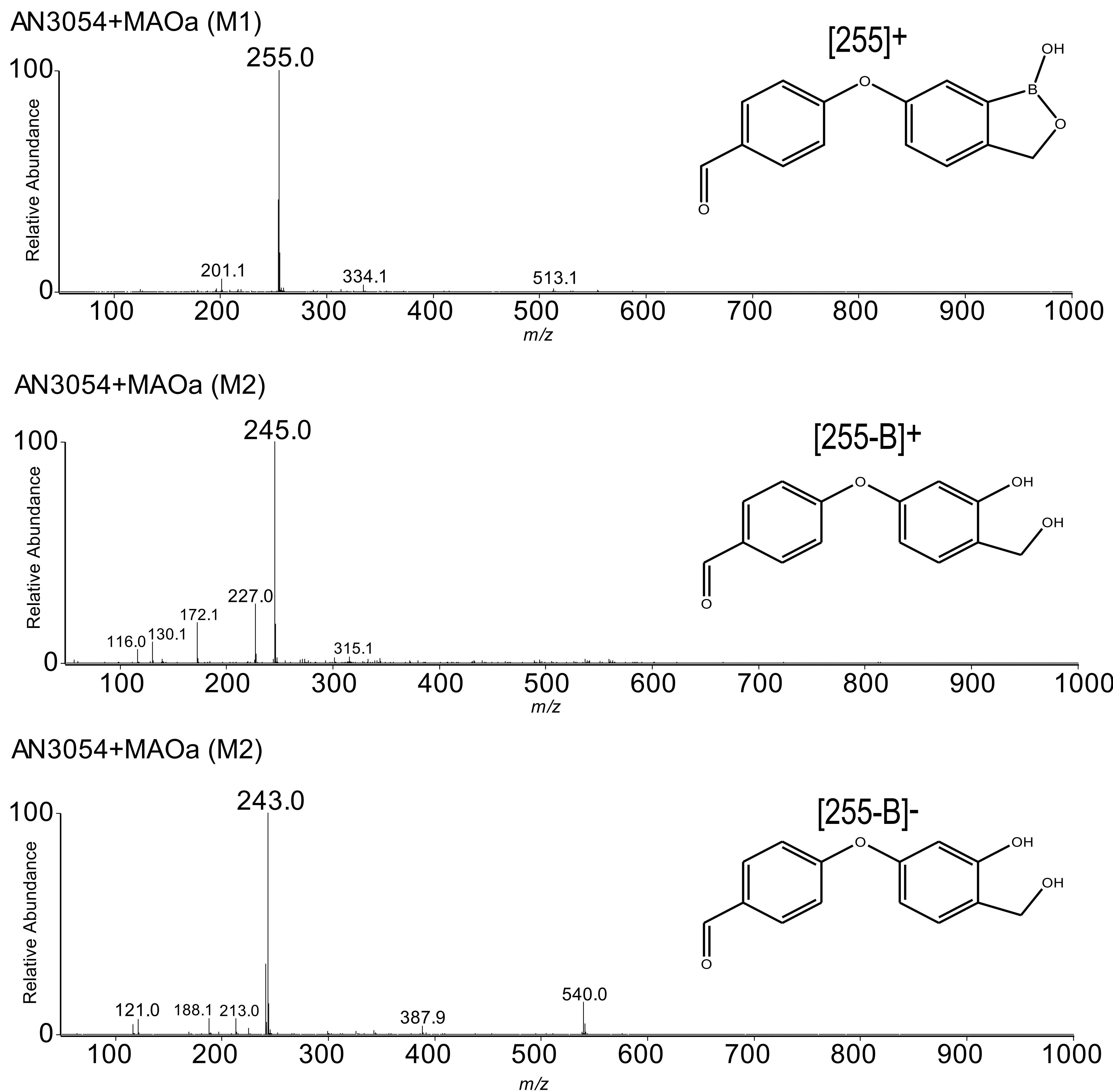

Supplement: S4 Fig — (A) HPLC peaks are indicated with corresponding retention time (RT), m/z of ion precursors identified in MS, and proposed structural formula. See S5 Fig for the detailed MS data. (B) MS spectrums of the ion precursors from either AN3054 or the metabolite from AN3054+TbALDH3. The [M+H]+ ion at m/z 239.0 was identified with both, indicating no change in structure. (C) MS spectrums of the ions precursors from either AN3330 or the metabolites from AN3054+MAOa+TbALDH3. The [M-H]- ion at m/z 269.0 was identified with both AN3330 and M-A1 metabolite, indicating an identical structure, which is consistent with detecting the [M+H]+ ion at m/z 271.0 from M-A1. The [M-H]- ion at m/z 259.0 was identified with M-A2 metabolite, resulted from the loss of boron, consistent with detecting the [M-H2O]+ ion at m/z 243.0 from M-A2. (d) MS spectrums of the ions precursors from the metabolites derived from AN3054+MAOa. The [M+H]+ ion at m/z 255.0 was identified with M1 metabolite, suggesting a conversion from methylamine-aldehyde occurred. The [M+H]+ ion at m/z 245.0 was identified from M2, indicating the loss of boron, consistent with detecting the [M-H]- ion at m/z 243.0. (PDF) [file ppat.1006850.s004.pdf]

TbALDH3 C<sub>259</sub>S  
+NAD +Mg<sup>2+</sup>

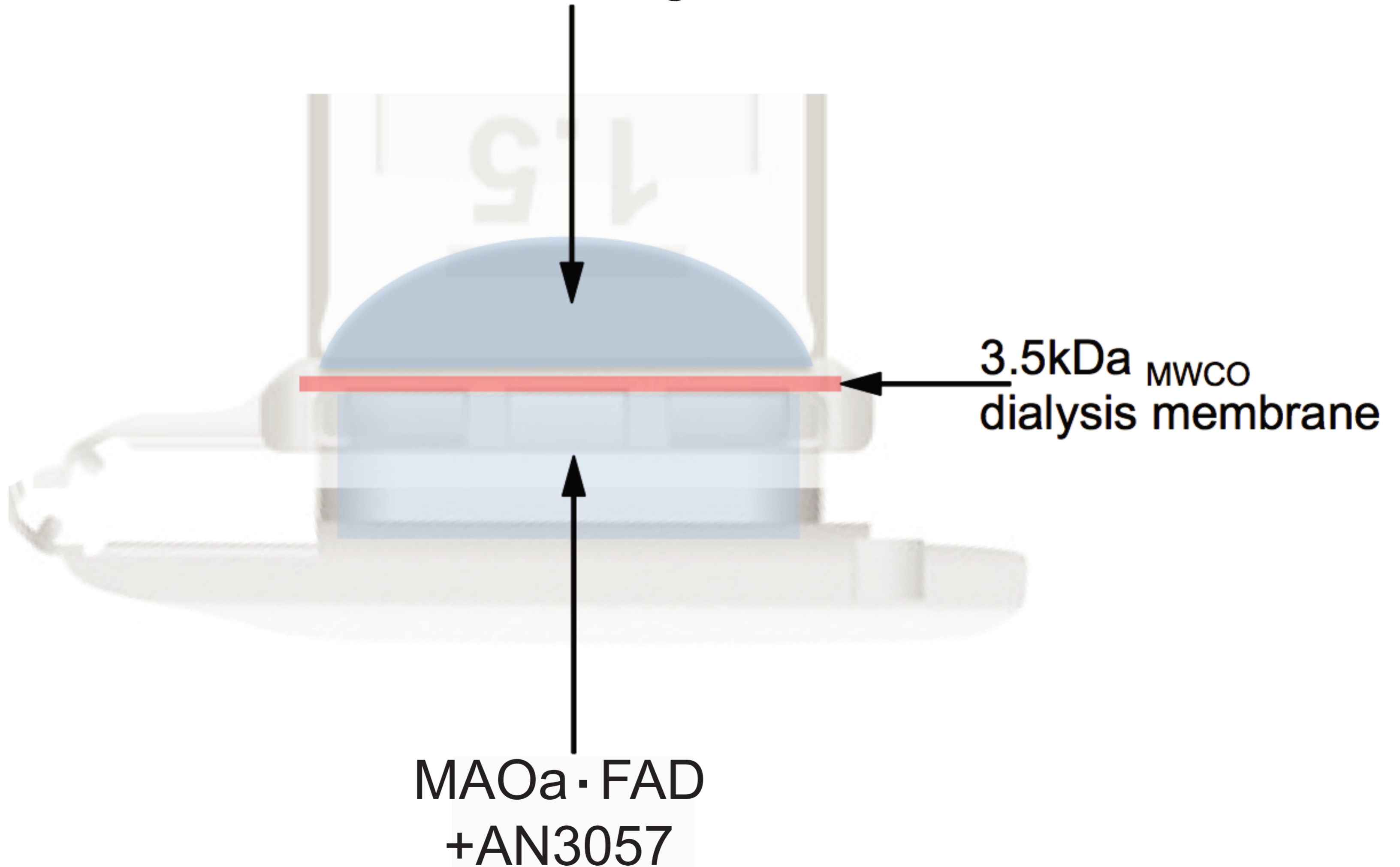

Supplement: S5 Fig — A 3.5 kDa dialysis membrane separates the two reservoirs allowing the diffusion of the benzaldehyde-benzoxaborole intermediate once formed by MaoA (lower reservoir) and subsequent binding by the catalytically inactive TbAlDH3 C259S mutant. (PDF) [file ppat.1006850.s005.pdf]

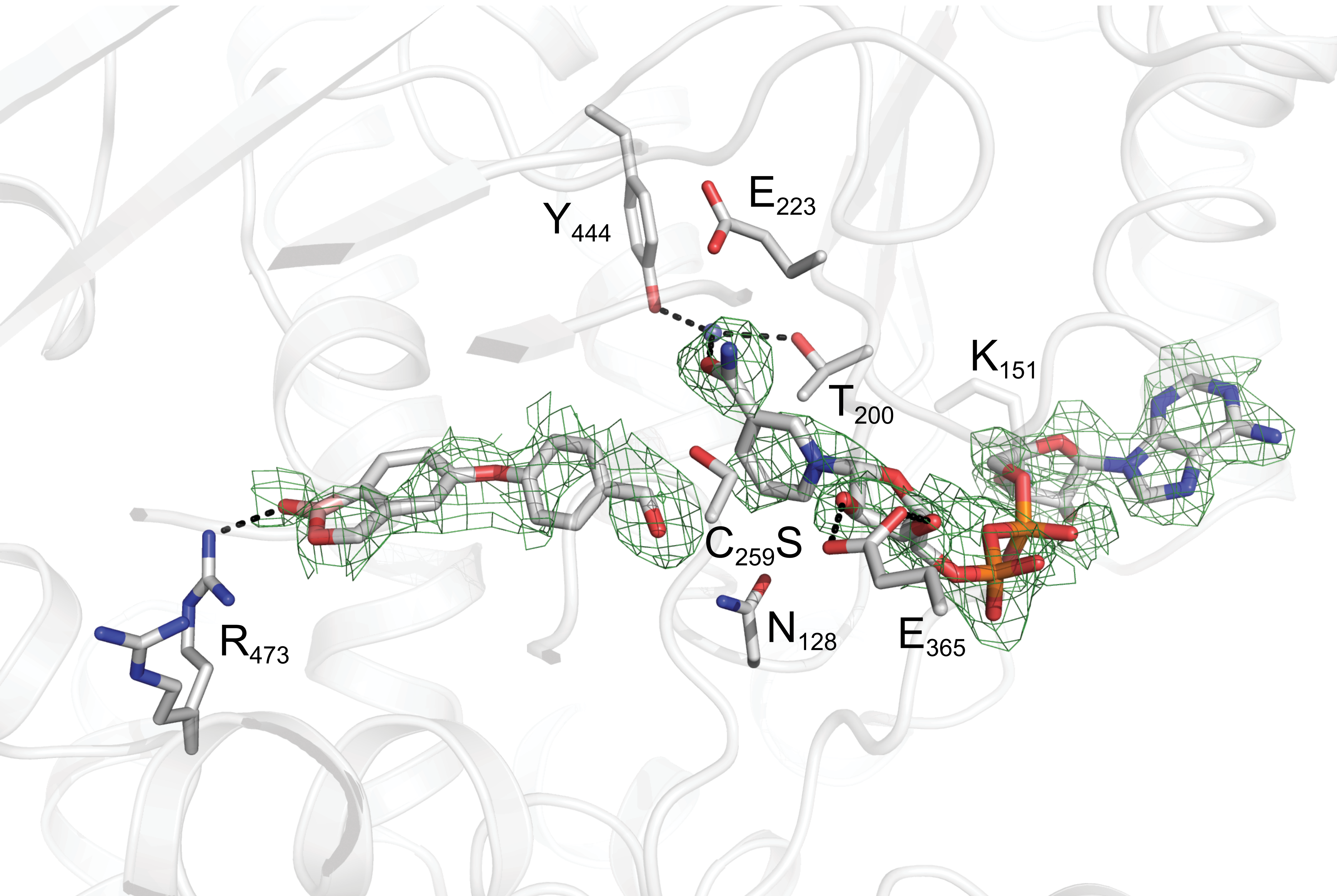

Supplement: S6 Fig — All key residues are in stick representation, in which non-carbon atoms are marked in color, oxygen in red, nitrogen in blue, and phosphorus in orange. Potential hydrogen bonds are depicted as black dashed lines;; selected water molecules as blue spheres. (PDF) [file ppat.1006850.s006.pdf]

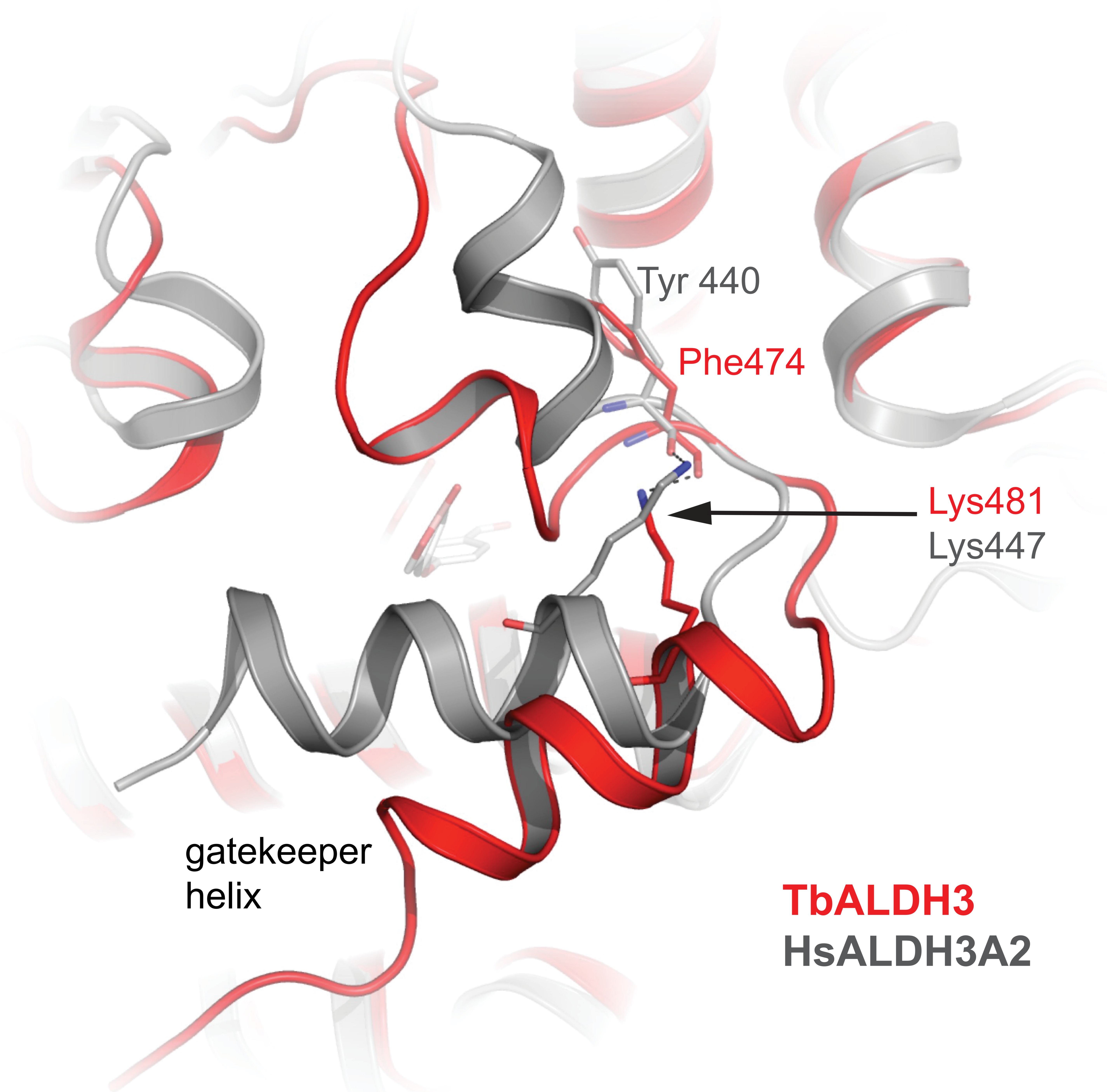

Tyr 440

Phe474

Lys481

Lys447

gatekeeper  
helix

**TbALDH3**

**HsALDH3A2**

Supplement: S8 Fig — Structural superposition in cartoon representation of TbALDH3 (red) with HsALDH3A2 (grey) focussing on the gatekeeper helix and the conserved hydrogen bonding interaction (dashed line) between K481 and the backbone carbonyl of F474. (PDF) [file ppat.1006850.s008.pdf]

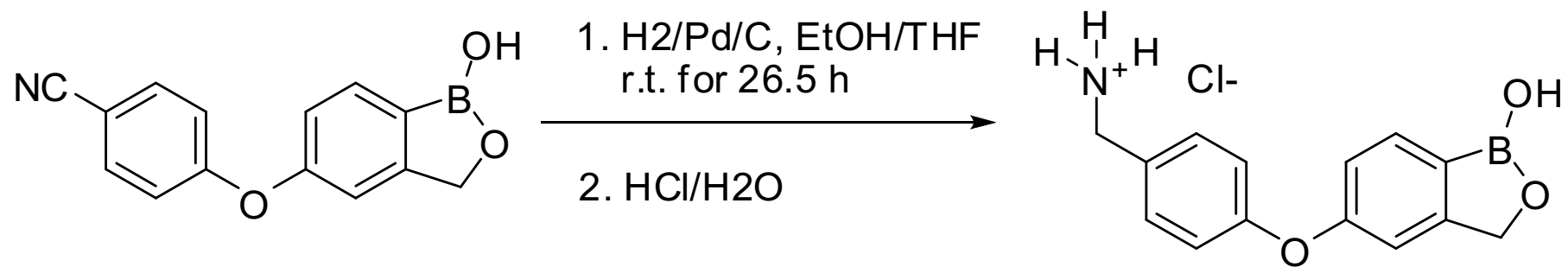

Supplement: S10 Fig — To the solution of 4-(1-hydroxy-1,3-dihydrobenzo[c][1,2]oxaborol-5-yloxy)benzonitrile (1.2 g, 4.78 mmol) in EtOH (150 mL) under N2 was added Pd/C (10 wt.%, 0.178 g). The reaction mixture was hydrogenated for 26.5 h using a H2 balloon at room temperature with stirring. The mixture was filtered, rotary evaporated and purified by silica gel column eluted with MeOH containing 0.6%volume NH4OH (3 mL 28–30% NH4OH to 500 mL MeOH). The white solid obtained was dissolved in water (80 mL) and 6N HCl (2 mL) was added, filtered and the filtrate was lyophilized to give the desired salt (4-(1-hydroxy-1,3-dihydrobenzo[c][1,2]oxaborol-5-yloxy)phenyl)methanaminium chloride as white solid (0.93 g, 3.19 mmol, yield 66.7%). M.p. > 250°C. 1H-NMR (DMSO-d6, 300 MHz): δ 9.18 (s, 1H), 8.43 (br. s, 3H), 7.74 (d, J = 8.1 Hz, 2H), 7.52 (d, J = 8.7 Hz, 2H), 7.08 (d, J = 8.7 Hz, 1H), 6.98–6.94 (m, 2H), 4.91 (s, 2H) and 3.99 (br. q, J = 4.8 Hz, 2H) ppm. Purity (HPLC): 94.9% at 254 nm. MS: m/z = 256 (M+1, ESI+) and m/z = 255 (M-, ESI-). (PDF) [file ppat.1006850.s010.pdf]
