# Supplementary material for: Host-parasite co-metabolic activation of antitrypanosomal aminomethyl-benzoxaboroles
Source: PLoS Pathog. 2018 Feb 9;14(2):e1006850. doi: 10.1371/journal.ppat.1006850 (PMC5823473; doi:10.1371/journal.ppat.1006850)
Supplement: S3 Fig — (A) MS spectrums of the ion precursors from either AN3057 or the metabolite from AN3057+TbALDH3. The [M+H]+ ion at m/z 239.0 was identified with both, indicating no change in structure. (B) MS spectrums of the ions precursors from either AN2861 or the metabolites from AN3057+MAOa+TbALDH3. The [M-H]- ion at m/z 269.0 was identified with both AN2861 and M-A1 metabolite, indicating an identical structure, which is consistent with detecting the [M+H]+ ion at m/z 271.0 from M-A1. The [M-H]- ion at m/z 259.0 was identified with M-A2 metabolite, resulted from the loss of boron, consistent with detecting the [M-H2O]+ ion at m/z 243.0 from M-A2. (C) MS spectrums of the ions precursors from the metabolites derived from AN3057+MAOa. The [M+H]+ ion at m/z 255.0 was identified with M1 metabolite, suggesting a methylamine- aldehyde conversion occurred. The [M+H]+ ion at m/z 245.0 was identified from M2, indicating the loss of boron, consistent with detecting the [M-H]- ion at m/z 243.0. (PDF) [file ppat.1006850.s003.pdf]

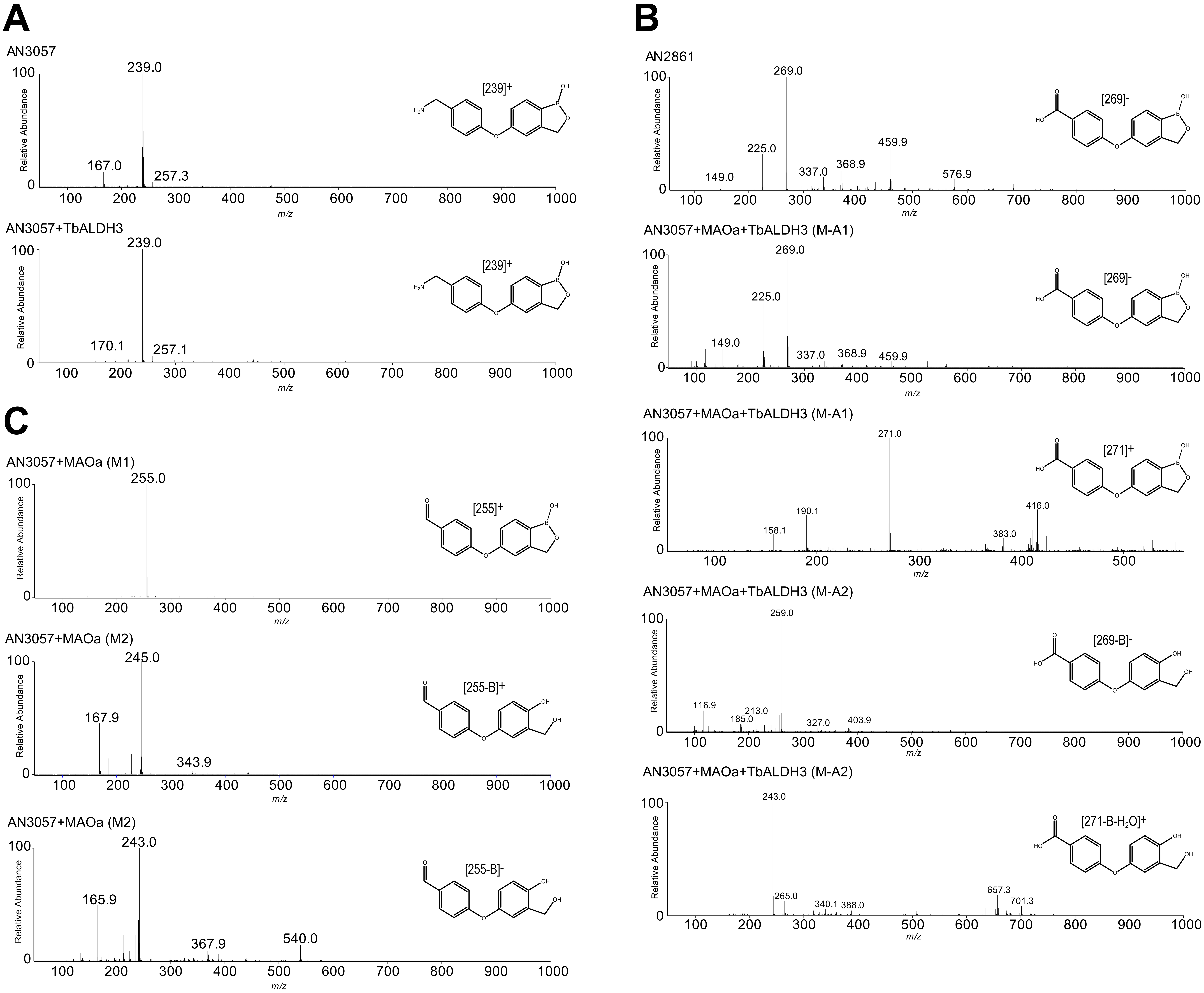
A
AN3057
[239]+
239.0
167.0
257.3
Relative Abundance
m/z
AN3057+TbALDH3
[239]+
239.0
170.1
257.1
B
AN2861
[269]-
269.0
225.0
149.0
337.0
368.9
459.9
576.9
AN3057+MAOa+TbALDH3 (M-A1)
[269]-
269.0
225.0
149.0
337.0
368.9
459.9
AN3057+MAOa+TbALDH3 (M-A1)
[271]+
271.0
158.1
190.1
383.0
416.0
AN3057+MAOa+TbALDH3 (M-A2)
[269-B]-
259.0
116.9
185.0
213.0
327.0
403.9
AN3057+MAOa+TbALDH3 (M-A2)
[271-B-H2O]+
243.0
265.0
340.1
388.0
657.3
701.3
C
AN3057+MAOa (M1)
[255]+
255.0
AN3057+MAOa (M2)
[255-B]+
245.0
167.9
343.9
AN3057+MAOa (M2)
[255-B]-
243.0
165.9
367.9
540.0
