# Supplementary material for: Host-parasite co-metabolic activation of antitrypanosomal aminomethyl-benzoxaboroles
Source: PLoS Pathog. 2018 Feb 9;14(2):e1006850. doi: 10.1371/journal.ppat.1006850 (PMC5823473; doi:10.1371/journal.ppat.1006850)
Supplement: S7 Fig — from T. brucei and H. sapiens (HsALDH3A1, P30838;; HsALDH3A2, P51648;; HsALDH3B1, P43353;; HsALDH3B2, P48448). Secondary structure elements are indicated for TbALDH3A2 (colored by domain: blue, catalytic domain; red, NAD-binding domain; green, oligomerisation domain) and HsALDH3A2 (colored in grey, from pdb 4QGK). The predicted C-terminal transmembrane segment in HsALDH3A2 is indicated and the prenylation/palmitoylation sites in HsALDH3B1 (Cys463 and Cys465) are highlighted in yellow. Note that the respective region in TbALDH3A2 does not to exhibit predicted transmembrane propensities and is lacking cysteine residues for lipidation. (PDF) [file ppat.1006850.s007.pdf]

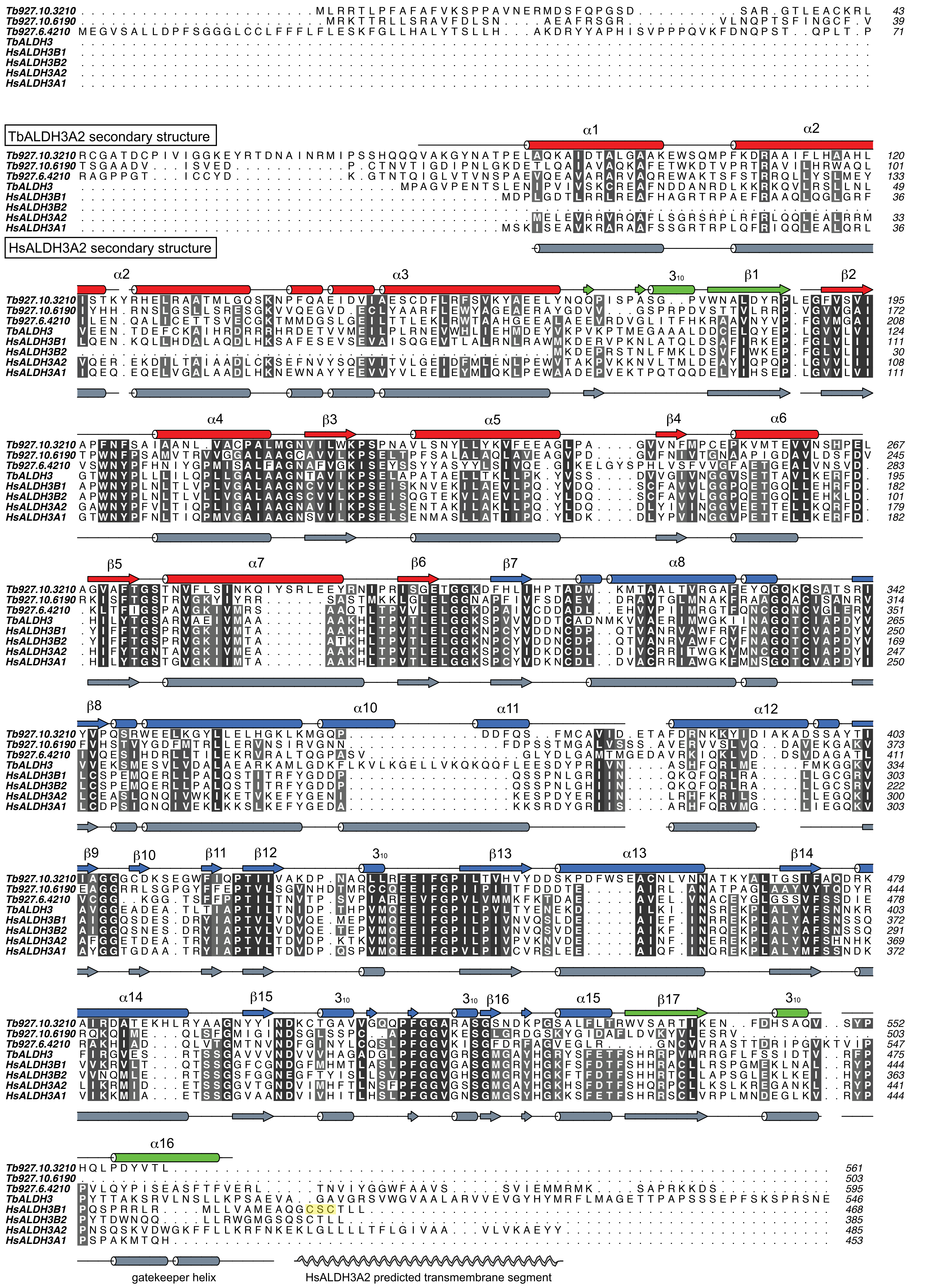

TbALDH3A2 secondary structure
HsALDH3A2 secondary structure
Tb927.10.3210
Tb927.10.6190
Tb927.6.4210
TbALDH3
HsALDH3B1
HsALDH3B2
HsALDH3A2
HsALDH3A1
α1
α2
α3
α4
α5
α6
α7
α8
α10
α11
α12
α13
α14
α15
α16
β1
β2
β3
β4
β5
β6
β7
β8
β9
β10
β11
β12
β13
β14
β15
β16
β17
gatekeeper helix
HsALDH3A2 predicted transmembrane segment
