# Supplementary material for: Host-parasite co-metabolic activation of antitrypanosomal aminomethyl-benzoxaboroles
Source: PLoS Pathog. 2018 Feb 9;14(2):e1006850. doi: 10.1371/journal.ppat.1006850 (PMC5823473; doi:10.1371/journal.ppat.1006850)
Supplement: S1 Table — (PDF) [file ppat.1006850.s011.pdf]

S1 Table TbALDH3 crystallography and refinement statistics

**Dataset**	**5MYP (wild type)**	**5NNO (C259S)**
Spacegroup	$P12_11$	$P12_11$
Wavelength (Å)	1.54178	1.54178
Resolution (Å)	90.70-1.95 (2.00-1.95)[a]	46.56-2.50 (2.60-2.50)[a]
Unit cell (Å)	87.72 63.95 91.75	89.17 62.52 92.97
Unique reflections	72557 (4318)	34306(3730)
Completeness (%)	99.4 (95.9)	97.6 (93.9)
<I/s(I)>	10.1 (3.8)	12.9 (8.0)
Multiplicity	4.9 (3.5)	3.1 (2.6)
R_{merge}[b]	0.099 (0.213)	0.074 (0.112)
R_{meas}	0.111 (0.250)	0.090 (0.138)
CC(1/2)	0.993 (0.941)	0.989 (0.968)
**Refined model**		
R_{work}[c]	0.20	0.24
R_{free}[d]	0.22	0.28
Protein residues	973	978
Other components	520 water,<br>1 glycerol	200 water, 2 NAD,<br>2 AN3057aldehyde
rmsd from ideal geometry		
bond lengths (Å)	0.007	0.011
bond angles (^{o})	1.01	1.55
Thermal parameter (*B*) values (Å^2)		
From Wilson plot	14.8	10.8
Mean *B* over all atoms	19.0	11.6
Mean *B* over all atoms for protein/waters/NAD/AN3057 aldehyde	19.1/23.6/35.0	26.2/14.4/21.5/27.1
Ramachandran favoured/allowed/outliers (%)	98.2 /1.2 /0.4	97.5/2.3/0.2

a. Values in parentheses refer to the highest resolution bin; **b.** $R_{merge} = \sum h \sum i |I(h,i) - \langle I(h) \rangle \sum h \sum i\ I(h,i)$; **c.** $R_{work} = \sum hkl||F_o|-|F_c||/\sum|F_o|$, where F_o is the observed structure factor amplitude and the F_c is the structure-factor amplitude calculated from the model; **d.** R_{free} is the same as R_{work} except only calculated using a subset, 5%, of the data that are not included in any refinement calculations.
