# Supplementary material for: Host-parasite co-metabolic activation of antitrypanosomal aminomethyl-benzoxaboroles
Source: PLoS Pathog. 2018 Feb 9;14(2):e1006850. doi: 10.1371/journal.ppat.1006850 (PMC5823473; doi:10.1371/journal.ppat.1006850)
Supplement: S2 Table — (PDF) [file ppat.1006850.s012.pdf]

S2 Table HPLC-MS analysis of AN3057-derived metabolites from MAOa-TbALDH3

**Peak ID**	**Tentative Metabolite Identification**	**Retention Time (min) Acidic eluent – basic eluent**
AN3057	Parent	2.38 - 2.39
M-A1	Acid	3.91 - 2.02
M-A2	Acid - diol	3.22 - 1.71
M1	Aldehyde	4.37 - 4.11
M2	Aldehyde - diol	3.61 - 3.61
