# Supplementary material for: Host-parasite co-metabolic activation of antitrypanosomal aminomethyl-benzoxaboroles
Source: PLoS Pathog. 2018 Feb 9;14(2):e1006850. doi: 10.1371/journal.ppat.1006850 (PMC5823473; doi:10.1371/journal.ppat.1006850)
Supplement: S3 Table — (PDF) [file ppat.1006850.s013.pdf]

S3 Table Metabolism profiles of *T. brucei* with the treatment of different benzoxaboroles

Metabolite	AN3057 LogFC (adj p value)	AN5568 LogFC (adj p value)
S-adenosylmethionine	2.36 (0.0011)	2.68 (0.0018)
Methylthioadenosine	2.87 (0.00044)	3.29
Methionine	0.2	-0.04
Adenine	1.36 (0.0087)	2.55 (0.0018)
Methyllysine	0.74	0.85
Dimethyllysine	0.6	1.08
Trimethyllysine	0.96	1.83 (0.03)

Note: The log2 fold change (LogFC) compared to time-matched DMSO control for each metabolite is shown. For metabolites with significant differences (adjusted p value of less than 0.05), the p-value is also shown adjusted by Benjamini-Hochberg procedure.
