# Supplementary material for: Host-parasite co-metabolic activation of antitrypanosomal aminomethyl-benzoxaboroles
Source: PLoS Pathog. 2018 Feb 9;14(2):e1006850. doi: 10.1371/journal.ppat.1006850 (PMC5823473; doi:10.1371/journal.ppat.1006850)
Supplement: S4 Table — (PDF) [file ppat.1006850.s014.pdf]

S4 Table Primers and construsts used in the work

**Strain**	**Gene ID**	**Construct**	**Primer-Forward**
TbALDH3 RNAi	Tb927.6.3050	pRPaiSL TbALDH3	5’-GTACGGATCCTCTAGACCAGTGATGCAGGAGGAAAT-3’
TbALDH3-6Myc	Tb927.6.3050	pNAT-TbALDH3-6Myc	5’-GATCTCTAGAATGCCAGCAGGTGTTCCGGAG-3’
TbALDH3	Tb927.6.3050	pET27bTbALDH3	5’-GCGCCCATGGCACCAGCAGGTGTTCCGGAGAATACC -3’
TbALDH3C256S	Tb927.6.3050	pET27bTbALDH3	5’-TCAACGCAGGTCAAACATCTATAGCGCCTGATTACGTTG-3’
