# Supplementary material for: Host-parasite co-metabolic activation of antitrypanosomal aminomethyl-benzoxaboroles
Source: PLoS Pathog. 2018 Feb 9;14(2):e1006850. doi: 10.1371/journal.ppat.1006850 (PMC5823473; doi:10.1371/journal.ppat.1006850)
Supplement: S5 Table — (PDF) [file ppat.1006850.s015.pdf]

S5 Table Species, strains and protein acessions inclued in tl

Species	Strain
Drosophila melanogaster	Release 6 ISO1
Caenorhabditis elegans	Bristol
Batracochytrium dendrobatidis	JAM
Aspergillus nidulans	FGS
Saccharomyces cerevisiae	ATCC 204508/S2
Schizosaccharomices pombe	972/ATCC 24
Leishmania brazliensis	MHOM/BR/75/M2904
Leishmania donovani	BPK28A1
Leishmania infantum	JPCM5
Leishmania major	Friedlin
Leishmania mexicana	MHOM/GT/2001/U1103
Leishmania tarentolae	Parrot-TarII
Leptomonas pyrrhocoris	H10
Trypanosoma brucei	TREU927
Trypanosoma vivax	Y486
Trypanosoma congolense	IL3000
Trypanosoma evansi	STIB805
Trypanosoma cruzi Sylvo	X10/1
Trypanosoma grayi	ANR4
Trypanosoma rangeli	SC58

lued in the phylogenetic analyses

Protein	Uniprot ID
ALDH1A1	P00352
ALDH1A2	O94788
ALDH1A3	P47895
ALDH1B1	P30837
ALDH1L1	O75891
ALDH1L2	Q3SY69
ALDH2	P05091
ALDH3A1	P30838
ALDH3A2	P51648
ALDH3B1	P43353
ALDH3B2	P48448
ALDH4A1	P30038
ALDH5A1	P51649
ALDH6A1	Q02252
ALDH7A1	P49419
ALDH8A1	Q9H2A2
ALDH9A1	P49189
ALDH16A	Q8IZ83
ALDH18A1	P54886
